# Regioselective and Stereoselective Epoxidation of n-3 and n-6 Fatty Acids by Fungal Peroxygenases

**DOI:** 10.3390/antiox10121888

**Published:** 2021-11-25

**Authors:** Alejandro González-Benjumea, Dolores Linde, Juan Carro, René Ullrich, Martin Hofrichter, Angel T. Martínez, Ana Gutiérrez

**Affiliations:** 1 Instituto de Recursos Naturales y Agrobiología de Sevilla (IRNAS), Consejo Superior de Investigaciones Científicas (CSIC), Reina Mercedes 10, E-41012 Seville, Spain; a.g.benjumea@irnas.csic.es; 2 Centro de Investigaciones Biológicas “Margarita Salas” (CIB), Consejo Superior de Investigaciones Científicas (CSIC), Ramiro de Maeztu 9, E-28040 Madrid, Spain; lolalinde@cib.csic.es (D.L.); jcarro@cib.csic.es (J.C.); 3 International Institute Zittau (IHIZ), Technische Universität Dresden, Markt 23, D-02763 Zitau, Germany; rene.ullrich@tu-dresden.de (R.U.); martin.hofrichter@tu-dresden.de (M.H.)

**Keywords:** unspecific peroxygenases, epoxylipids, polyunsaturated fatty acids, omega 3 (n-3) fatty acids, omega 6 (n-6) fatty acids, bioactive compounds, regioselective synthesis, stereoselective synthesis, NMR, chiral HPLC

## Abstract

Epoxide metabolites from n-3 and n-6 polyunsaturated fatty acids arouse interest thanks to their physiological and pharmacological activities. Their chemical synthesis has significant drawbacks, and enzymes emerge as an alternative with potentially higher selectivity and greener nature. Conversion of eleven eicosanoid, docosanoid, and other n-3/n-6 fatty acids into mono-epoxides by fungal unspecific peroxygenases (UPOs) is investigated, with emphasis on the *Agrocybe aegerita* (*Aae*UPO) and *Collariella virescens* (r*Cvi*UPO) enzymes. GC-MS revealed the strict regioselectivity of the n-3 and n-6 reactions with *Aae*UPO and r*Cvi*UPO, respectively, yielding 91%-quantitative conversion into mono-epoxides at the last double bond. Then, six of these mono-epoxides were obtained at mg-scale, purified and further structurally characterized by ^1^H, ^13^C and HMBC NMR. Moreover, chiral HPLC showed that the n-3 epoxides were also formed (by *Aae*UPO) with total *S/R* enantioselectivity (*ee* > 99%) while the n-6 epoxides (from r*Cvi*UPO reactions) were formed in nearly racemic mixtures. The high regio- and enantioselectivity of several of these reactions unveils the synthetic utility of fungal peroxygenases in fatty acid epoxidation.

## 1. Introduction

Epoxylipids include a series of epoxide metabolites from n-3 and n-6 (also known as omega 3 and omega 6) polyunsaturated eicosanoid and docosanoid acids, among others, that play important roles in a range of biological processes [1]. These compounds are mediators in the inflammatory response [2] and are also involved in the regulation of blood pressure [3], pain perception [4], and angiogenesis [5]. Moreover, epoxylipids are strongly linked to the endothelial system as well as to organs such as heart and kidney, contributing to controlling hypertension and cardiovascular diseases [6,7,8].

Epoxides of polyunsaturated fatty acids—such as arachidonic (AA), eicosapentaenoic (EPA) and docosahexaenoic acids (DHA)—are naturally synthesized by cytochrome P450 monooxygenases (P450s) as animal metabolites, delivering them in a range of regioisomers. These *cis*-epoxides can subsequently be hydrolyzed to vicinal diols by epoxide hydrolases making the study of their biological activities difficult. Moreover, the above epoxides can be synthesized as a pair of *R*/*S* and *S*/*R* enantiomers from the two distinguishable faces of the substrate double bond (*re*,*si* and *si*,*re*).

Non-enzymatic synthesis of the above compounds with peracids, such as *m*-chloroperbenzoic acid, generally yields complex mixtures of mono-epoxides, together with minor amounts of other epoxides, that make the synthesis scarcely effective for obtaining individual epoxides. Other (semi)synthetic approaches have been explored [9,10], but they have the drawbacks of involving several steps, with low overall yields and use of high-cost and hazardous reagents. The enzymatic synthesis by P450 enzymes (EC 1.14) emerged as an alternative to classical chemical synthesis supported by its higher regio- and stereoselectivities, P450 BM3 from *Bacillus megaterium* and its variants being the most studied enzymes [11,12]. Plant peroxygenases have also been used for these enzymatic syntheses [13,14,15].

Unspecific peroxygenases (UPOs, EC 1.11.2.1) are a (super)family of mono(per)oxygenases firstly described for *Agrocybe aegerita* cultures in 2004 [16] that, in contrast to the above P450s, only require H_2_O_2_ for activation and function, as other peroxygenases. UPOs have demonstrated to catalyze a great repertoire of oxygenation reactions discussed in several reviews [17,18,19,20,21,22]. Additionally, epoxidation of complex mixtures of unsaturated free fatty acids and their methyl esters from vegetable oils have been recently reported for UPOs [23]. The advantages of UPOs, compared with P450, are related with the use of H_2_O_2_ (or organic hydroperoxides) as the only co-substrate, and with a better stability thanks to their extracellular nature, while compared to plant peroxygenases UPOs have the advantage of their microbial origin facilitating the enzyme production. The above characteristics turned UPOs into powerful tools for synthetic applications to obtain a variety of added value oxyfunctionalized compounds [19,22].

Although thousands of putative UPO genes have been identified in genomes [24] since the discovery of *A. aegerita* UPO (*Aae*UPO), only a few additional UPO enzymes are available from: (i) fungal wild-type cultures—such as those of the basidiomycetes *Coprinellus radians* [25], *Marasmius rotula* [26] and *Marasmius wettsteinii* [27], and the ascomycete *Chaetomium globosum* [28]; and (ii) heterologous expression of UPO genes from *Coprinopsis cinerea* (r*Cci*UPO) [29] and *Humicola insolens* [28] in *Aspergillus oryzae*, *M. rotula* (r*Mro*UPO) [30], *Collariella virescens* (r*Cvi*UPO) and *Daldinia caldariorum* [31] in *Escherichia coli*, and an evolved *Aae*UPO [32,33], whose mutations have been also exported for expressing two other UPOs [34], in yeast. However, a modular yeast secretion system has been recently claimed for the universal heterologous production of UPOs [35,36]. The above heterologous expression systems allowed the design of mutated variants that spread and improve the performance of native UPOs [30,37,38,39,40].

The present work describes the selective conversion of representative n-3 and n-6 polyunsaturated fatty acids (i.e., those with the last double bond located at the third and sixth positions from the end of the molecule, respectively) to the corresponding mono-epoxides by *Aae*UPO and r*Cvi*UPO, which were selected in an initial screening also including r*Cci*UPO and r*Mro*UPO. The substrate conversion and regioselectivity of the enzymatic reactions were evaluated by GC-MS. Furthermore, mono-epoxides from six fatty acids were obtained at mg-scale, purified, and their structures confirmed by ^1^H, ^13^C and HMBC NMR. Finally, chiral HPLC analyses were performed to determine the enantioselectivity of the *Aae*UPO and r*Cvi*UPO reactions.

## 2. Materials and Methods

### 2.1. Chemicals

**Figure 1:**
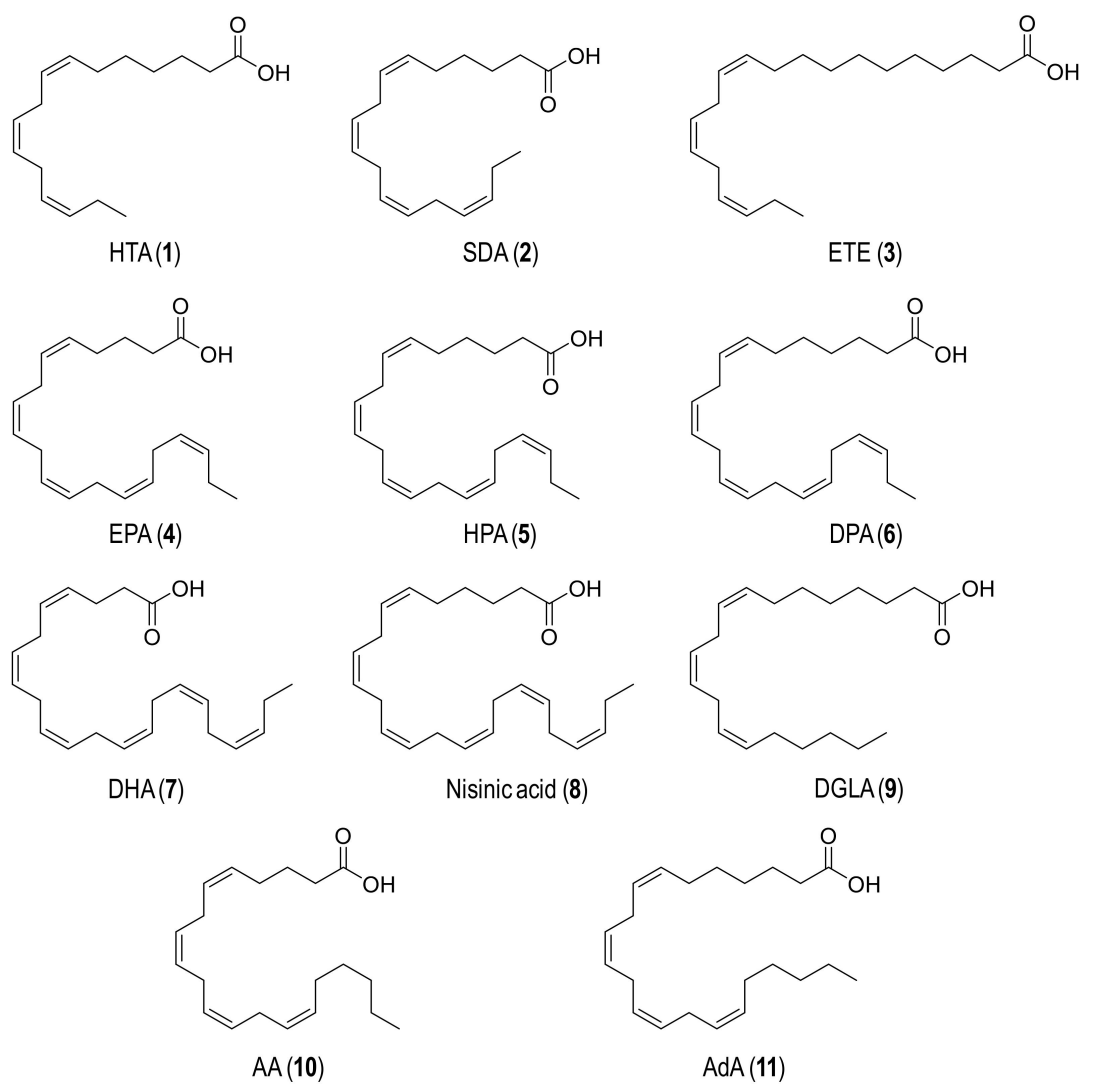
Formulae of the n-3 (**1**–**8**) and n-6 (**9**–**11**) fatty acids used in enzymatic epoxidation reactions: (**1**) Hexadeca-7,10,13-trienoic acid (HTA); (**2**) Octadeca-6,9,12,15-tetraenoic acid (SDA); (**3**) Eicosa-11,14,17-trienoic acid (ETE); (**4**) Eicosa-5,8,11,14,17-pentaenoic acid (EPA); (**5**) Heneicosa-6,9,12,15,18-pentaenoic acid (HPA); (**6**) Docosa-7,10,13,16,19-pentaenoic acid (DPA); (**7**) Docosa-4,7,10,13,16,19-hexaenoic acid (DHA); (**8**) Tetracosa-6,9,12,15,18,21-hexaenoic acid (nisinic acid); (**9**) Eicosa-8,11,14-trienoic acid (DGLA); (**10**) Eicosa-5,8,11,14-tetraenoic acid (AA); and (**11**) Docosa-7,10,13,16-tetraenoic acid (AdA).

Eleven n-3 (**1**–**8**) and n-6 (**9**–**11**) polyunsaturated fatty acids (Figure 1) were tested as substrates for UPO epoxidation. These compounds −namely hexadeca-7,10,13-trienoic acid (HTA; **1**), octadeca-6,9,12,15-tetraenoic acid (stearidonic acid, SDA; **2**), eicosa-11,14,17-trienoic acid (ETE; **3**), eicosa-5,8,11,14,17-pentaenoic acid (EPA; **4**), heneicosa-6,9,12,15,18-pentaenoic acid (HPA; **5**), docosa-7,10,13,16,19-pentaenoic acid (DPA; **6**), docosa-4,7,10,13,16,19-hexaenoic acid (DHA; **7**), tetracosa-6,9,12,15,18,21-hexaenoic acid (nisinic acid; **8**), eicosa-8,11,14-trienoic acid (dihomo-γ-linoleic acid, DGLA; **9**), eicosa-5,8,11,14-tetraenoic acid (AA; **10**) and docosa-7,10,13,16-tetraenoic acid (adrenic acid, AdA; **11**)− were supplied by Larodan (*cis* isomers in all cases).

Mono-epoxide standards −namely (±)17,18-epoxyeicosa-5,8,11,14-tetraenoic acid (17,18-EpETrE), (±)19,20-epoxydocosa-7,10,13,16-tetraenoic acid (19,20-EpDTrE), (±)19,20-epoxydocosa-4,7,10,13,16-pentaenoic acid (19,20-EpDPE), (±)14,15-epoxyeicosa-5,8,11-trienoic acid (14,15-EpETE) and 14(*S*),15(*R*)-epoxyeicosa-5,8,11-trienoic acid (14(*S*),15(*R*)-EpETE)—were supplied by Cayman (*cis* isomers in all cases). The GC/HPLC-derivatizing reagents BSTFA ([*N*,*O*-bistrimethylsilyl]trifluoroacetamide) and trimethylsilyldiazomethane were obtained from Merck.

### 2.2. Enzymes

*Aae*UPO is a wild-type enzyme obtained from cultures of the agaric basidiomycete *A. aegerita* TM-A1 in soybean-peptone medium, and purified as described elsewhere [24]. Recombinant r*Cci*UPO was supplied by Novozymes A/S and corresponds to the protein model 7249 from the genome of *C. cinerea* sequenced at the DOE JGI (http://genome.jgi.doe.gov/Copci1, accessed on 19 October 2021) expressed in *A. oryzae* (patent WO/2008/119780). Recombinant r*Mro*UPO and r*Cvi*UPO enzymes were produced by expressing the corresponding gene sequences—from Gröbe et al. [26] and Lund et al. [41], respectively—in *E. coli* as active cytosolic proteins, and purified as reported previously [30,39]. Site-directed mutagenesis production of the F88L and T158F variants of r*Cvi*UPO has also already been reported [41]. In all cases, enzyme concentrations were spectrophotometrically estimated from the spectra of the reduced-UPO adducts with CO [42].

### 2.3. Enzymatic Reactions

The enzymes—namely *Aae*UPO (50 pmol), r*Cci*UPO (25–50 pmol), r*Mro*UPO (50–100 pmol), r*Cvi*UPO and its variants (100 pmol)—were added to a solution of the substrate (0.1 μmol) in 1 mL of phosphate, pH 7 (pH 5.5 for r*Mro*UPO), containing 20% acetone. The solution was heated to 30 °C and the reaction triggered by adding H_2_O_2_ (initial and 4 subsequent pulses up to 1 µmol). After 30-min reaction, the products were recovered with methyl *tert-*butyl ether and derivatized with BSTFA (80 °C, 1 h) for GC-MS analyses as trimethylsilyl derivatives, as described below.

For preparative synthesis, the reactions were carried out following the procedures described above but in larger volumes (50–500 mL) and purified by silica-gel (60–200 μm) column chromatography with hexane-EtOAc (10:1→1:5) as mobile phase. The isolated products were further characterized by chiral HPLC (after methylation with trimethylsilyl diazomethane for 1 h at room temperature) and NMR, as described below.

### 2.4. GC-MS Analyses

GC-MS analyses were performed with a Shimadzu GC-MS QP2020 Ultra equipment using a DB-5HT (30 m × 0.25 mm i.d. and 0.1 μm film thickness) capillary column. Helium was used as carrier gas at a flow of 0.83 mL·min^−1^. The injection was performed at 300 °C, the oven was heated from 120 °C (1 min) to 300 °C (15 min) at a rate of 5 °C·min^−1^ and the transfer line was set at 300 °C. Compounds were identified by mass fragmentography and by comparison with authentic standards. Quantification was carried out from total-ion peak areas, after deconvolution (with Origin, 2020, software) when partial overlapping was produced.

### 2.5. HPLC Analyses

Chiral analyses of the mono-epoxides purified as described above were performed with a Shimadzu i-Prominence 2030C equipment using an OB-H (250 × 4.6 cm, 5 μm particle) column. Compounds were eluted with hexane-isopropanol-AcOH (99.65:0.3:0.05), at a flow rate of 1 mL·min^−1^, and monitored at 202 or 204 nm depending on the maximal absorbance of the epoxide product. Authentic standards were used for assigning retention times.

### 2.6. NMR Analyses

**Figure 2:**
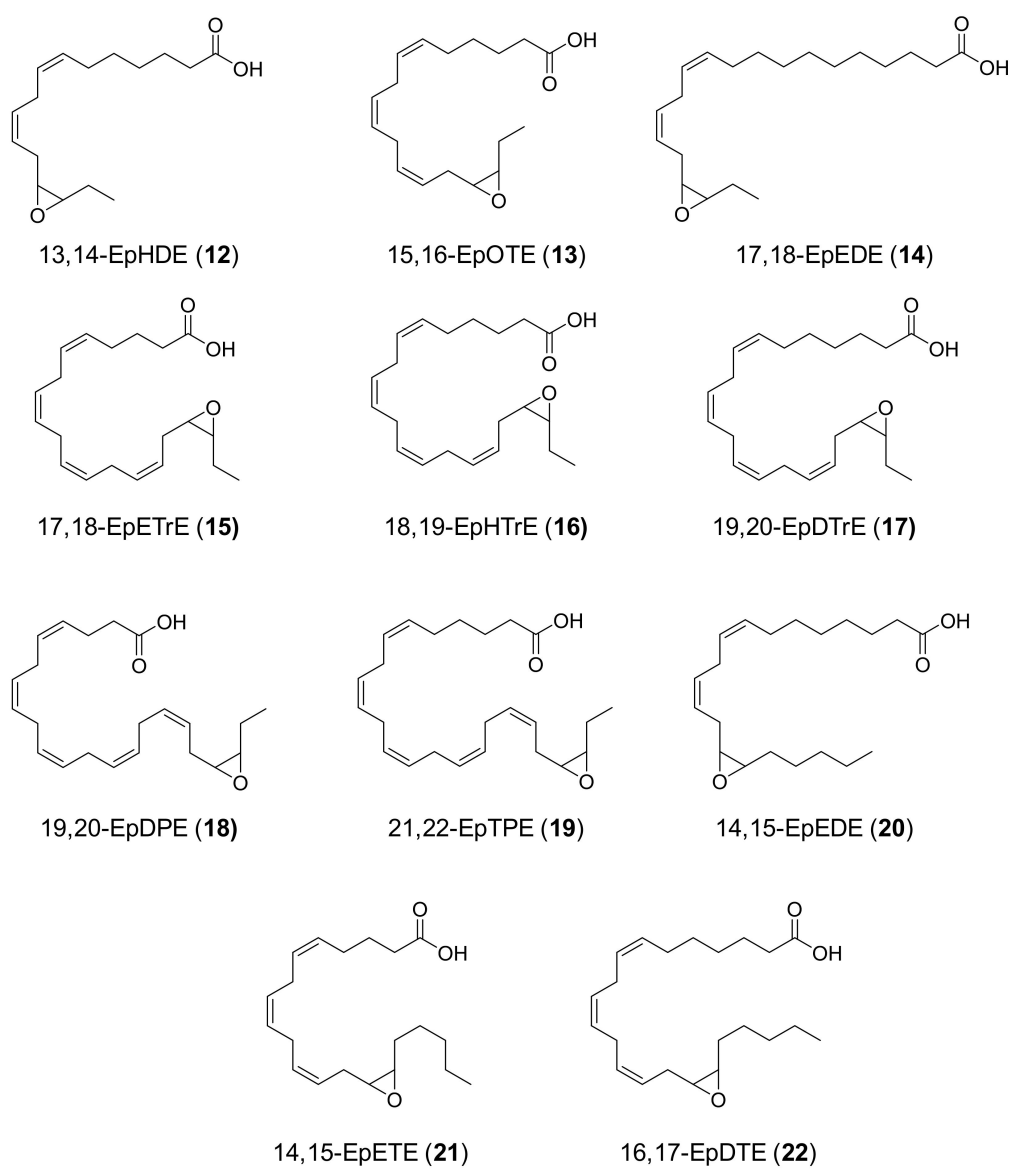
Formulae of main products (**12**–**22**) from UPO epoxidation of n-3/n-6 fatty acids (Figure 1): (**12**) 13,14-epoxyhexadeca-7,10-dienoic acid (13,14-EpHDE); (**13**) 15,16-epoxyoctadeca-6,9,12-trienoic acid (15,16-EpOTE); (**14**) 17,18-epoxyeicosa-11,14-dienoic acid (17,18-EpEDE); (**15**) 17,18-epoxyeicosa-5,8,11,14-tetraenoic acid (17,18-EpETrE); (**16**) 18,19-epoxyheneicosa-6,9,12,15-tetraenoic acid (18,19-EpHTrE); (**17**) 19,20-epoxydocosa-7,10,13,16-tetraenoic (19,20-EpDTrE); (**18**) 19,20-epoxydocosa-4,7,10,13,16-pentaenoic acid (19,20-EpDPE); (**19**) 21,22-epoxytetracosa-6,9,12,15,18-pentaenoic acid (21,22-EpTPE); (**20**) 14,15-epoxyeicosa-8,11-dienoic acid (14,15-EpEDE); (**21**) 14,15-epoxyeicosa-5,8,11-trienoic acid (14,15-EpETE); and (**22**) 16,17-epoxydocosa-7,10,13-trienoic acid (16,17-EpDTE).

1D and 2D NMR analyses were carried out with a Bruker AVIII 500 MHz equipment coupled with a cryoprobe. The ^1^H and ^13^C NMR chemical shifts (δ, ppm), calibrated using the signal of residual non-deuterated chloroform (at 7.26 and 77.16 ppm, respectively), and the coupling constants (*J*, Hz) of the six mono-epoxides analyzed (Figure 2, compounds **14, 15, 17, 18, 20** and **21**) are provided below.

17,18-Epoxyeicosa-11,14-dienoic acid (17,18-EpEDE; **14**). ^1^H-NMR (CDCl_3_, 500 MHz; Figure S3 top) δ: 5.53–5.29 (m, 4H, H-11, H-12, H-14, H-15), 2.97 and 2.91 (2dt, 1H each, *J*_17,18_ = 4.2 Hz, *J*_17,16_ = 6.4 Hz, *J*_18,19_ = 6.3 Hz, H-17, H-18), 2.80 (t, 2H, *J* = 7.0 Hz, H-13), 2.42 (dt, 1H, *J*_16a,16b_ = 15.0 Hz, *J* = 6.3 Hz, H-16a), 2.34 (t, 2H, *J* = 7.6 Hz, H-2), 2.22 (dt, 1H, *J*_16b,16a_ = 15.0 Hz, *J* = 6.9 Hz, H-16b), 2.04 (dt, 2H, *J* = 7.0 Hz, *J* = 6.9 Hz, H-10), 1.66–1.49 (m, 4H, H-3, H-19), 1.34–1.27 (m, 12H, H-4, H-5, H-6, H-7, H-8, H-9), 1.05 (t, 3H, *J* = 7.6 Hz, H-20) ppm. ^13^C-NMR (CDCl_3_, 125 MHz; Figure S3 bottom) δ: 179.0 (C-1), 131.0, 130.8, 127.6 and 124.3 (C-11, C-12, C-14, C-15), 58.6 (C-18), 56.8 (C-17), 34.0 (C-2), 29.7, 29.6, 29.5, 29.4, 29.3, 29.2, 27.4, 26.3, 26.0, 24.8 (C-3, C-4, C-5, C-6, C-7, C-8, C-9, C-10, C-13, C-16), 21.2 (C-19). 10.7 (C-20) ppm.

17,18-EpETrE (**15**). ^1^H-NMR (CDCl_3_, 500 MHz; Figure S4 top) δ: 5.55–5.34 (m, 8H, H-5, H-6, H-8, H-9, H-11, H-12, H-14, H-15), 3.02 and 2.95 (2dt, 1H each, *J*_17,18_ = 4.3 Hz, *J*_17,16_ = 6.4 Hz, *J*_18,19_ = 6.3 Hz, H-17, H-18), 2.86–2.81 (m, 6H, H-7, H-10, H-13), 2.50–2.43 (m, 1H, H-16a), 2.38–2.34 (m, 2H, H-2), 2.22 (dt, 1H, *J*_16a,16b_ = 14.9 Hz, *J* = 7.3 Hz, H-16b), 2.14 (dt, 2H, *J* = 7.6 Hz, *J* = 6.9 Hz, H-4), 1.71 (q, 2H, *J* = 7.2 Hz, H-3), 1.66–1.52 (m, 2H, H-19), 1.05 (t, 3H, *J* = 7.4 Hz, H-20) ppm. ^13^C-NMR (CDCl_3_, 125 MHz; Figure S4 bottom) δ: 177.5 (C-1), 130.6 (C-14), 129.0, 128.8, 128.4, 128.3, 128.0 and 127.8 (C-5, C-6, C-8, C-9, C-11 and C-12), 127.3 (C-15), 58.7 (C-18), 56.8 (C-17), 33.0 (C-2), 27.0, 26.4, 26.1, 25.8, 25.7 and 24.5 (C-3, C-4, C-7, C-10, C-13 and C-16), 21.0 (C-19), 10.6 (C-20) ppm.

19,20-EpDTrE (**17**). ^1^H-NMR (CDCl_3_, 500 MHz; Figure S5 top) δ: 5.54–5.31 (m, 8H, H-7, H-8, H-10, H-11, H-13, H-14, H-16, H-17), 2.97 and 2.91 (2dt, 1H each, *J*_19,20_ = 4.2 Hz, *J*_19,18_ = 6.4 Hz, *J*_20,21_ = 6.3 Hz, H-19, H-20), 2.85–2.79 (m, 6H, H-9, H-12, H-15), 2.44-2.38 (m, 1H, H-18a), 2.34 (t, 2H, *J* = 7.5 Hz, H-2), 2.25–2.19 (m, 1H, H-18b), 2.06 (dt, 2H, *J* = 6.5 Hz, *J* = 6.6 Hz, H-6), 1.67–1.49 (m, 4H, H-3, H-21), 1.39–1.32 (m, 4H, H-4, H-5), 1.05 (t, 3H, *J* = 7.5 Hz, H-22) ppm. ^13^C-NMR (CDCl_3_, 125 MHz; Figure S5 bottom) δ: 178.6 (C-1), 130.6, 130.2, 128.7, 128.6, 128.0, 127.9 and 127.8 (C-7, C-8, C-10, C-11, C-13, C-14, C-16), 124.6 (C-17), 58.6 (C-20), 56.8 (C-19), 34.0 (C-2), 29.3, 28.8, 27.1, 26.3, 25.9, 25.8 and 24.7 (C-3, C-4, C-5 C-6, C-9, C-12, C-15, C-18), 21.2 (C-21), 10.7 (C-22) ppm.

19,20-EpDPE (**18**). ^1^H-NMR (CDCl_3_, 500 MHz; Figure S6 top) δ: 6.54–5.33 (m, 10H, H-4, H-5, H-7, H-8, H-10, H-11, H-13, H-14, H-16, H-17), 2.98 and 2.92 (2dt, 1H each, *J*_19,20_ = 4.2 Hz, *J*_19,18_ = 6.4 Hz, *J*_20,21_ = 6.3 Hz, H-19, H-20), 2.85–2.83 (m, 8H, H-6, H-9, H-12, H-15), 2.45–2.40 (m, 5H, H-2, H-3, H-18a), 2.25–2.19 (m, 1H, H-18b), 1.66–1.49 (m, 2H, H-21), 1.05 (t, 3H, *J* = 7.5 Hz, H-22) ppm. ^13^C-NMR (CDCl_3_, 125 MHz; Figure S6 bottom) δ: 178.0 (C-1), 130.6, 129.6, 128.5, 128.3, 128.2, 128.1, 128.0 and 127.8 (C-4, C-5, C-7, C-8, C-10, C-11, C-13, C-14, C-16), 124.5 (C-17), 58.7 (C-20), 56.9 (C-19), 34.0 (C-2), 26.2, 26.0, 25.8, 25.7, 25.6 and 22.7 (C-3, C-6, C-9, C-12, C-15, C-18, C-19), 21.1 (C-21), 10.7 (C-22) ppm.

14,15-Epoxyeicosa-8,11-dienoic acid (14,15-EpEDE; **20**). ^1^H-NMR (CDCl_3_, 500 MHz; Figure S7 top) δ: 5.53–5.31 (m, 4H, H-8, H9, H-11, H-12), 2.98–2.93 (m, 2H, H-14, H-15), 2.80 (t, 2H, *J* = 6.9 Hz, H-10), 2.41 (dt, 1H, *J*_13a,13b_ = 15.0 Hz, *J* = 6.3 Hz, H-13a), 2.35 (t, 2H, *J*_2,3_ = 7.5 Hz, H-2), 2.21 (dt, 1H, *J*_13b,13a_ = 15.0 Hz, *J* = 6.5 Hz, H-13b), 2.07–2.03 (m, 2H, H-7), 1.64 (q, 2H, *J* = 7.3 Hz, H-3), 1.57–1.48 (m, 3H, H-16a, H-17), 1.45–1.44 (m, 1H, H-16b), 1.38–1.30 (m, 10H, H-4, H-5, H-6, H-18, H-19), 0.90 (t, 3H, *J* = 7.3 Hz, H-20) ppm. ^13^C-NMR (CDCl_3_, 125 MHz; Figure S7 bottom) δ: 178.5 (C-1), 130.8 and 127.5 (C-8, C-12), 130.5 and 124.2 (C-9, C-11), 57.4 (C-15), 56.6 (C-14), 33.8 (C-2), 31.8 (C-18), 29.4, 28.8 (C-5, C-6), 28.9 (C-4), 27.7 (C-17), 27.2 (C-7), 26.3 (C-16), 26.2 (C-13), 25.9 (C-10), 24.7 (C-3), 22.6 (C-19), 14.0 (C-20) ppm.

14,15-EpETE (**21**). ^1^H-NMR (CDCl_3_, 500 MHz; Figure S8 top) δ: 5.57–5.34 (m, 6H, H-5, H-6, H-8, H-9, H-11, H-12), 3.00–2.95 (m, 2H, H-14, H-15), 2.86–2.80 (m, 4H, H-7, H-10), 2.48–2.40 (m, 1H, H-13a), 2.36 (t, 2H, *J*_2,3_ = 7.3 Hz, H-2), 2.28–2.19 (m, 1H, H-13b), 2.14 (c, 2H, *J* = 7.3 Hz, H-4), 1.72 (q, 2H, *J* = 7.4 Hz, H-3), 1.59–1.43 (m, 4H, H-16, H-17), 1.37–1.28 (m, 4H, H-18, H-19), 0.90 (t, 3H, *J* = 7.1 Hz, H-20) ppm. ^13^C-NMR (CDCl_3_, 125 MHz; Figure S8 bottom) δ: 177.9 (C-1), 130.7, 129.1, 129.0, 128.6, 127.8 and 124.4 (C-5, C-6, C-8, C-9, C-11 and C-12), 57.8 (C-15), 56.8 (C-14), 33.2 (C-2), 31.9 (C-18), 27.8 (C-16), 26.6 (C-4), 26.4 (C-17), 26.3 (C-13), 26.0 (C-7), 25.8 (C-10), 24.6 (C-3), 22.7 (C-19), 14.1 (C-20) ppm.

## 3. Results and Discussion

### 3.1. UPO Screening for Epoxidation of n-3 and n-6 Fatty Acids

**Figure 3:**
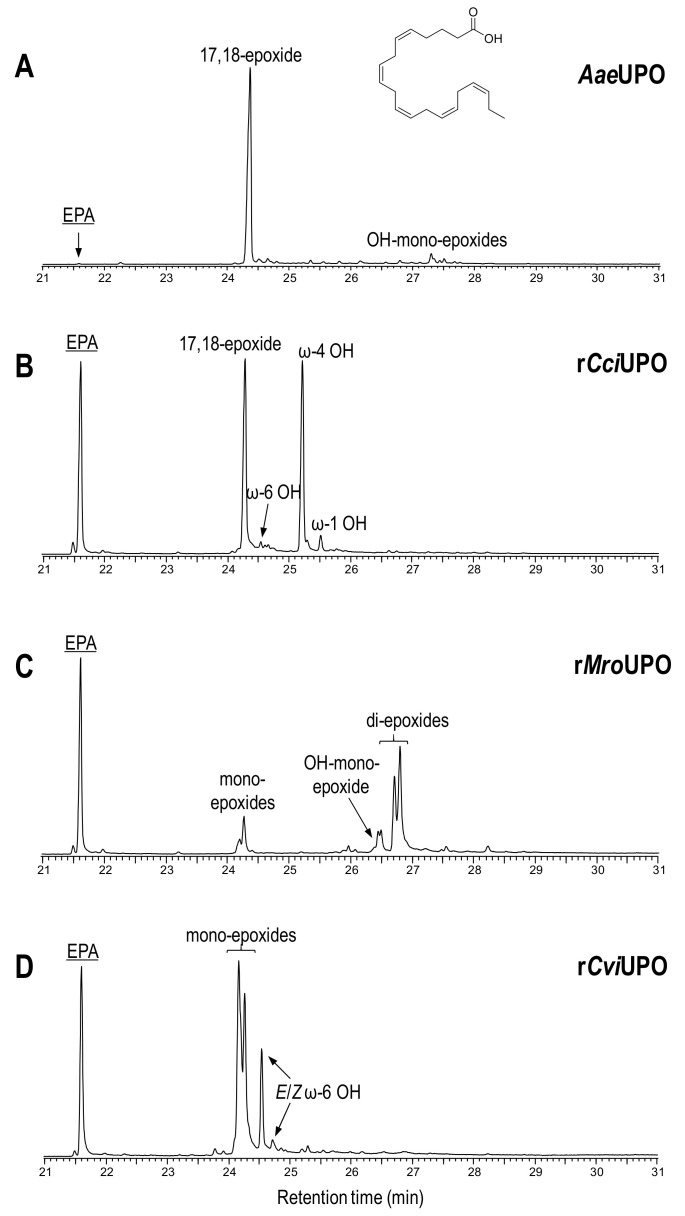
Oxyfunctionalization patterns of n-3 compound **4** (EPA) by different UPOs.

**Figure 4:**
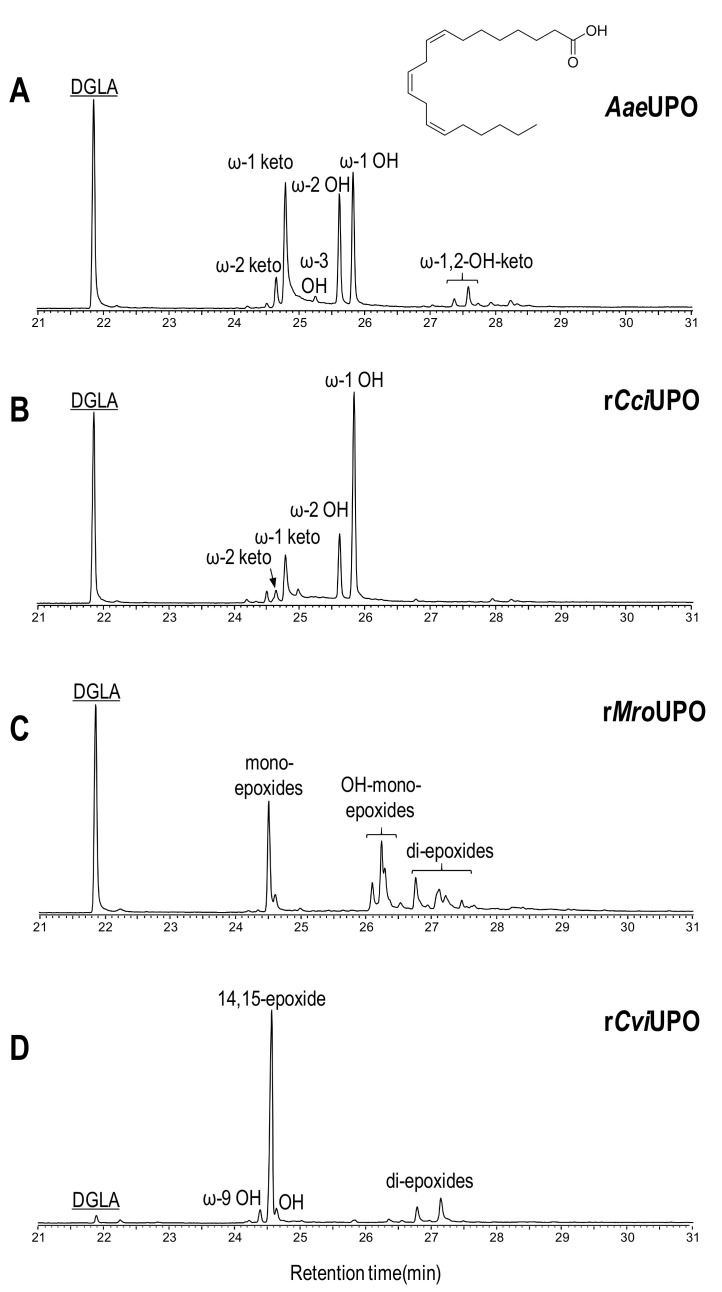
Oxyfunctionalization patterns of n-6 compound **9** (DGLA) by different UPOs.

For studying the enzymatic epoxidation patterns, EPA (**4**) and DGLA (**9**) were selected as model n-3 and n-6 fatty acids, respectively, and four fungal UPOs representative of the long—*Aae*UPO (353 aa) and *Cci*UPO (329 aa)—and short—*Mro*UPO (234 aa) and *Cvi*UPO (259 aa)—families [16] were screened (Figure 3 and Figure 4).

With EPA (**4**), *Aae*UPO (Figure 3A) exhibited the highest substrate conversion (lowest substrate peak at 21.6 min retention time) only yielding one mono-epoxide (tentatively 17-epoxide) and very small amounts of hydroxy-epoxides. The same mono-epoxide was formed by r*Cci*UPO (Figure 3B) but hydroxylated EPA derivatives were also observed (50% of the total products) mainly at the ω-4 position. Conversely, r*Mro*UPO (Figure 3C) produced mixtures of mono-epoxide and di-epoxide regioisomers, together with some hydroxy-epoxides. Likewise, r*Cvi*UPO (Figure 3D) caused unspecific mono-epoxidation, yielding at least 2 mono-epoxides, as well as one hydroxy fatty acid (at ω-6 position).

The enzymatic activity of *Aae*UPO with DGLA (**9**) considerably differed from the reaction with its n-3 counterpart, since only hydroxy and ketone derivatives (at ω-1 and ω-2 positions) were found (Figure 4A) and no epoxidation products were formed. These results reveal that epoxidation of subterminal double bond (n-3 position in EPA) is strongly preferred over alkyl chain hydroxylation at the same position (in DGLA).

r*Cci*UPO (Figure 4B) showed a similar oxygenation pattern to that of *Aae*UPO but with a higher proportion of the hydroxy derivative at ω-1 position. The oxygenation profile obtained with r*Mro*UPO (Figure 4C) was similar to that produced with EPA, mainly forming complex mixtures of mono-epoxides (including hydroxy-epoxides) and di-epoxides. Finally, high conversion and successful mono-epoxidation of DGLA (product tentatively assigned to the 14-epoxide) was accomplished in the reaction with r*Cvi*UPO (Figure 4D), where only negligible amounts of di-epoxides and hydroxy derivatives were observed.

The results of r*Mro*UPO reactions with both EPA and DGLA are in agreement with previous reports showing that this enzyme exhaustively epoxidizes polyunsaturated plant fatty acids, such as linoleic and α-linolenic acids, producing mixtures of mono- and di-epoxides [43]. Moreover, the oxygenation patterns by *Aae*UPO and r*Cci*UPO can be correlated with another previous work [38] where *Aae*UPO allowed the epoxidation of α-linolenic acid at the last double bond with strict regioselectivity.

**Figure 5:**
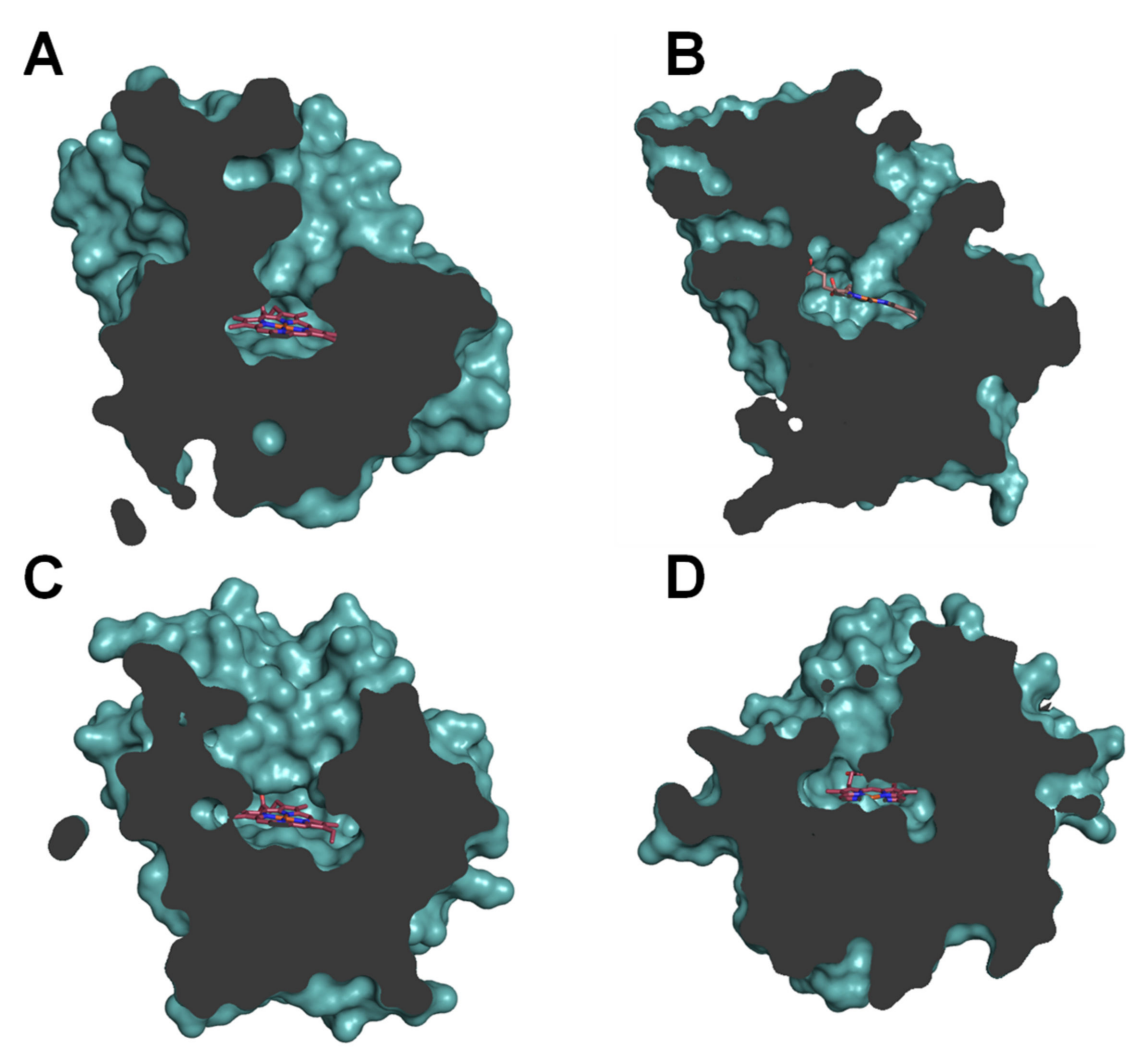
Sections of the *Aae*UPO (**A**), *Cci*UPO (**B**), *Mro*UPO (**C**) and *Cvi*UPO (**D**) molecules, highlighting the differences in heme access channels. External surface of the protein and surface of the residues lining the channel are shown in light teal, and the buried heme is displayed as sticks in CPK coloring. From PDB entries 2YP1 (**A**) and 5FUJ (**C**) and homology models (**B**,**D**).

These results can be partially rationalized in accordance with the topology of the heme-access channels (Figure 5). Under this assumption, narrow-channel UPOs, such as *Aae*UPO (Figure 5A) and r*Cci*UPO (Figure 5B), would only oxygenate at most terminal positions. Therefore, subterminal double bonds, as present in n-3 fatty acids, are epoxidized by *Aae*UPO since only their last double bond could attain the active site, whereas with n-6 acids it only produces subterminal hydroxy and keto derivatives (Figure 3A and Figure 4A). In contrast, the enzymatic activity that r*Mro*UPO exhibited with polyunsaturated fatty acids could be explained thanks to its wider access channel, which allows substrate bending and, thus, promotes EPA (**4**) diepoxidation (Figure 3C). Finally, r*Cvi*UPO turned out to be an intermediate between *Aae*UPO (and r*Cci*UPO) and r*Mro*UPO in terms of the access channel, resulting in non-specific mono-epoxidation of EPA (**4**)**,** which becomes highly regioselective with DGLA (**9**) (Figure 3D and Figure 4D, respectively). These findings reveal that *Aae*UPO and r*Cvi*UPO are good candidates for the selective synthesis of mono-epoxides at the last double bond of n-3 and n-6 unsaturated fatty acids, respectively, as described below.

### 3.2. Regioselective Synthesis of n-3 Fatty-Acid Mono-Epoxides by AaeUPO

The results reported in Section 3.1 prompted the study of up to eight n-3 compounds—namely HTA (**1**), SDA (**2**), ETE (**3**), EPA (**4**), HPA (**5**), DPA (**6**), DHA (**7**), and nisinic acid (**8**) (see Figure 1)— as substrates of *Aae*UPO. All these compounds yielded mono-epoxides at the last (n-3) double bond (products **12**–**19** in Figure 2) at high proportion (91–97% of products) in quantitative conversions, along with negligible amounts of other hydroxylated (epoxidized or not) compounds (Table 1) as revealed by the chromatographic analyses (Figure S1).

It is remarkable that the enzyme showed strict regioselectivity (>99% of products) towards compound **2** (SDA). Such epoxidation at the last double bond was confirmed by GC-MS using authentic standards when available, as in the cases of **15**, **17** and **18** products. Otherwise, regioselectivity was also tentatively assigned by mass fragmentography, due to the characteristic fragment originating from interaction of the carbonyl group and the open oxirane ring (Scheme S1) that appears at 217, 247, 243, 271, 269, and 297 *m*/*z* in the spectra of **13**–**15** and **17**–**19**, respectively.

**Figure 6:**
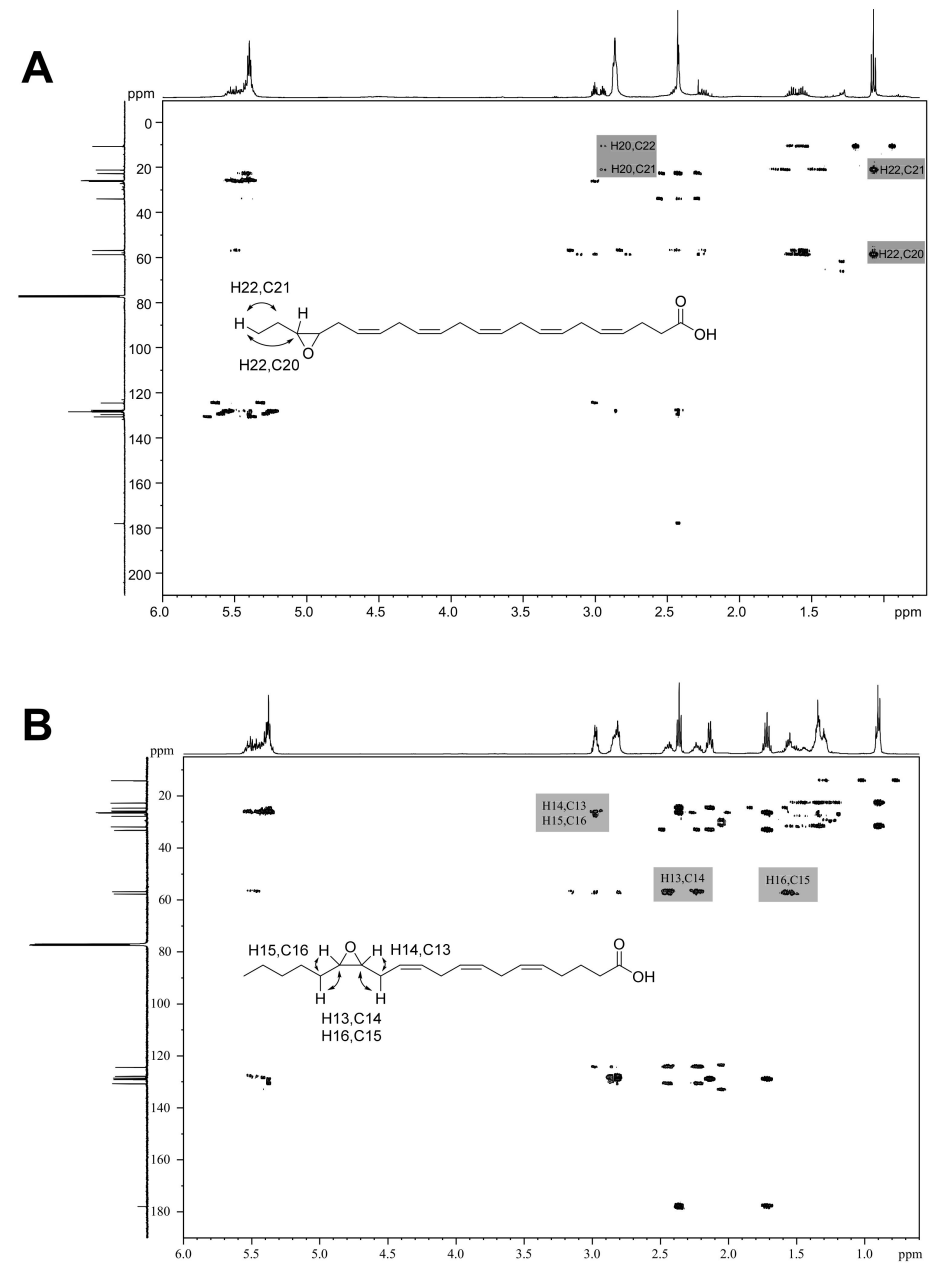
HMBC 2D-NMR spectra of enzymatic epoxides: (**A**) 19,20-EpDPE (**18**) from DHA (**7**) reaction with *Aae*UPO; and (**B**) 14,15-EpETE (**21**) from AA (**10**) reaction with r*Cvi*UPO. Several relevant correlation signals are indicated.

Then, the epoxides from easily available compounds **3**, **4**, **6** and **7** (products **14**, **15**, **17** and **18**) were synthesized with *Aae*UPO, isolated by column chromatography at mg-scale (68–77% yield), and characterized by NMR. The epoxidation at the last double bond was confirmed by 1D (Figures S3–S6) and 2D (Figures S9 and S10 top) NMR thanks to: (i) the shift downfield, from 0.97–0.98 to 1.05 ppm, of the triplet signal of the terminal methyl in the ^1^H-NMR spectrum, due to the induction effect of the oxirane ring; and (ii) the correlation signals observed in the HMBC spectra: *H*_20_-*C*_18_ and *H*_18_-*C*_19,20_ for eicosanoid acids, and *H*_22_-*C*_20_ and *H*_20_-*C*_21,22_ for docosanoid acids. Two examples of HMBC analyses of one n-3 and one n-6 epoxide are shown in Figure 6A,B, respectively (and additional HMBC spectra are provided in Figures S9 and S10).

Among n-3 epoxides, NMR spectra of **15** and **18** were already available in the literature [10,44], but those of compounds **14** and **17** were not previously reported. The regioselectivities observed in the *Aae*UPO reactions are the highest reported to date for these compounds. In different publications, P450 BM3 and its F87V variant have demonstrated to be capable of yielding mono-epoxides at the last double bond from **4** and **7,** but with lower regioselectivities [11,45,46].

### 3.3. Regioselective Synthesis of n-6 Fatty-Acid Mono-Epoxides by rCviUPO and Two Variants

In the light of the results reported in Section 3.1, three n-6 compounds —namely DGLA (**9**), AA (**10**) and AdA (**11**) (see Figure 1)— were studied as substrates of r*Cvi*UPO and its F88L and T158F variants, that differ in the size of the access channel [39]. These variants were preferred over the heme-channel variants previously generated for r*Mro*UPO [38] because of the much better heterologous expression of r*Cvi*UPO [39]. The r*Cvi*UPO reactions (Table 1) resulted in high conversions (94–99%) and yielded the mono-epoxides at the last double bond (compounds **20**–**22** in Figure 2) as the main products (74–92%) as revealed by the chromatographic analyses (Figure S2). Nevertheless, some hydroxy and/or di-epoxide derivatives were also observed, especially with DGLA and AdA.

In the F88L reactions (Figure S2B), the conversions were in the same range (95–99%) as with the wild-type enzyme (while T158F conversion of AA was comparatively low), but the amounts of di-epoxides were higher, especially with AdA (80% of the F88L reaction products). The above results are consistent with a wider heme access-channel in this variant (Figure S11B), enabling epoxidation at two different double bonds (most probably at n-9 in addition to n-6) yielding di-epoxides. On the other hand, with T158F the selectivity to form mono-epoxides was higher since no di-epoxides were detected (Figure S2C), in agreement with its narrowed heme channel (Figure S11C). In this sense, the r*Cvi*UPO T158F variant is reminiscent of the narrow-channel I153F/S156F variant of r*Mro*UPO [38], but the former has the advantage of its much better heterologous expression levels, as mentioned above.

In the case of n-6 fatty acids, identification of the mono-epoxide by GC-MS of an authentic standard could only be confirmed for **21**, as it is the only commercially available product. In order to unequivocally assign the regioselectivity of mono-epoxidation in the n-6 reactions, the epoxides from **9** and **10** (compounds **20** and **21**) were enzymatically synthesized, isolated at mg-scale (63% and 66% yield, respectively) and characterized by NMR spectroscopy. Spectra of the n-6 epoxides compounds **20** and **21** were already available in the literature [47,48]. Indeed, HMBC correlations between the oxirane ring and the surrounding nucleus were observed for both **20** and **21** epoxides, which unambiguously confirmed the regioselective epoxidation, as illustrated in Figure 6B (and Figure S10 bottom).

As mentioned for n-3 fatty acids, the P450 BM3 enzyme and its F87V variant (since wild-type BM3 has the disadvantage of also introducing subterminal hydroxyls) have been used in the synthesis of mono-epoxides of polyunsaturated fatty acids. Among n-6 fatty acids, only **10** (arachidonic acid) showed total regioselectivity for the 14,15-epoxide in P450 reactions [49], although with low substrate conversion.

**Scheme 1:**
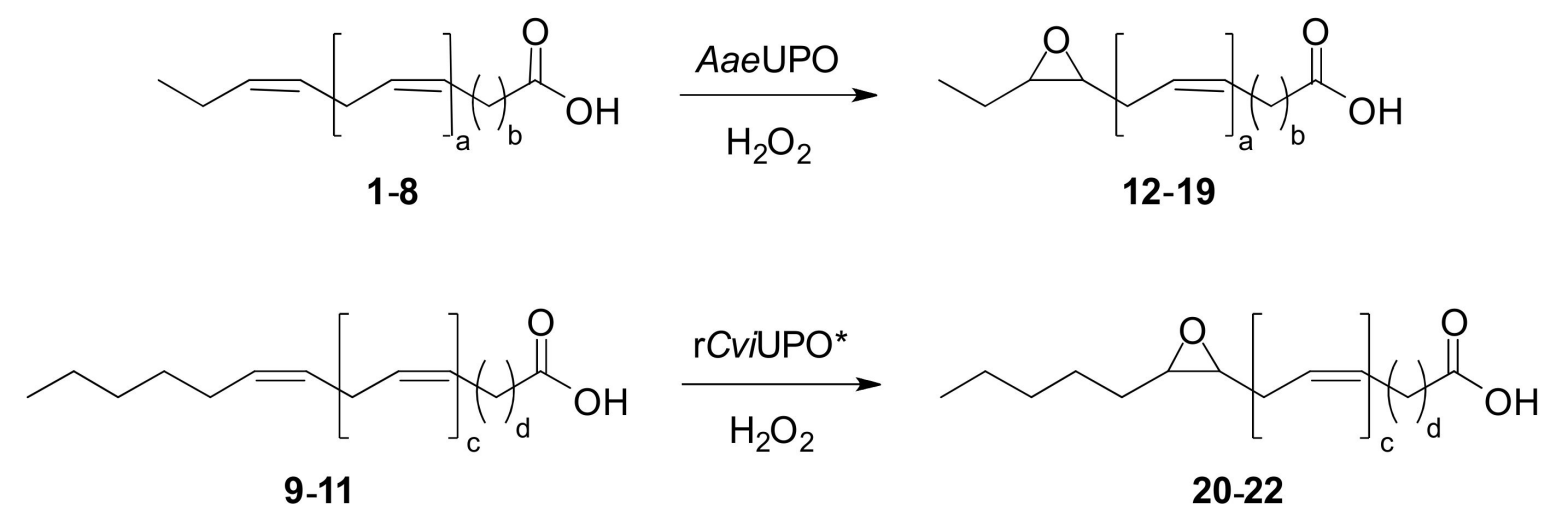
Characteristic enzymatic mono-epoxidation patterns of n-3 and n-6 polyunsaturated fatty acids by *Aae*UPO and r*Cvi*UPO, respectively. * Also including the r*Cvi*UPO T158F variant.

The different regioselectivities of *Aae*UPO and r*Cvi*UPO in mono-epoxidation of n-3 and n-6 fatty acids are illustrated in Scheme 1 top and bottom, respectively.

### 3.4. Stereoselectivity of Mono-Epoxides from n-3 and n-6 Fatty Acids

**Figure 7:**
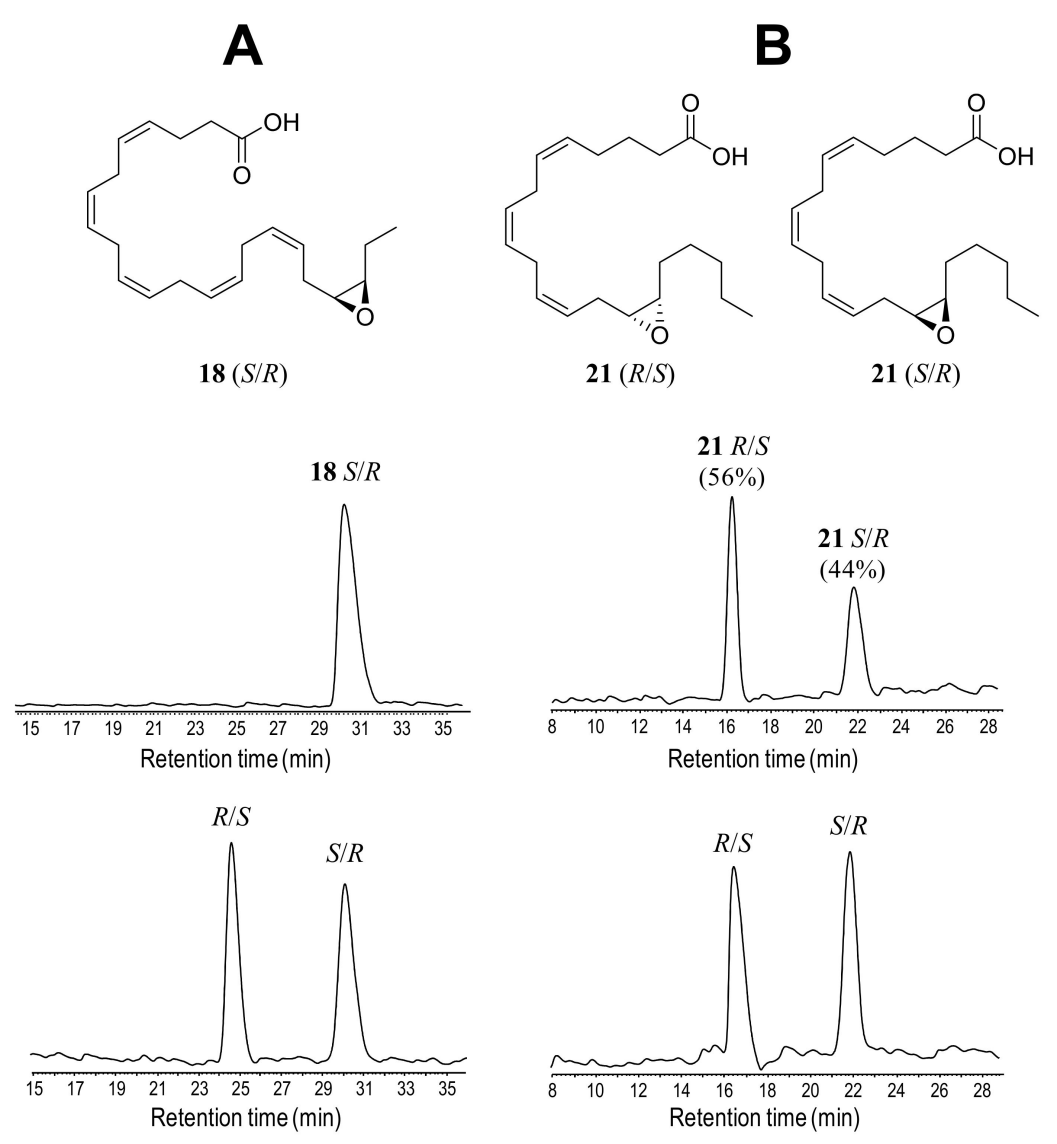
Chiral HPLC profiles (at 202 nm) and formulae of isolated n-3 and n-6 mono-epoxides: (**A**) 19(*S*),20(*R*)-EpDPE (**18**) from *Aae*UPO reaction with DHA (**7**) (top) compared with a racemic standard (bottom); and (**B**) 14(*R*),15(*S*)-EpETE and 14(*S*),15(*R*)-EpETE (**21**) from r*Cvi*UPO reaction with AA (**10**) (top) compared with a racemic standard (bottom).

Animated by the high regioselectivity of the enzymatic mono-epoxidation reactions, the stereoselectivity of the products was analyzed by chiral HPLC. These analyses were performed with compounds **14**, **15**, **17**, **18**, **20** and **21**, after production at mg-scale with 74%, 68%, 71%, 77%, 63% and 66% yields, respectively, and derivatization into methyl esters. For product identification, racemic standards of **15**, **17**, **18** and **21** were separated. The elution order of the enantiomers was assigned: (i) as described in the literature for n-3 epoxides (analyzed with the same chiral column) after determining the absolute configuration of epoxide **15 [11]**, which was extended to other n-3 mono-epoxides [49]; and (ii) by comparison with the commercially available 14(*S*),15(*R*)-EpETE for n-6 epoxides. In general, *R*/*S* enantiomers in racemic standards (Figure 7A,B and Figure S12A–D, bottom) eluted at lower retention times than *S*/*R* enantiomers under the chromatographic conditions used.

Chiral analysis of the *Aae*UPO reactions with n-3 fatty acids (Figure 7A, Figure S12A–C top, and S12E) revealed that the mono-epoxides were selectively produced as *S*/*R* enantiomers exclusively (Table 2). Conversely, the mono-epoxides from n-6 fatty acids (Figure 7B, Figures S12D top and S12F,) were synthesized with low enantioselectivity, as shown by the reactions with r*Cvi*UPO and its T158F variant yielding in both cases 56% of the 14(*S*),15(*R*)-EpEDE (**20**) (tentative enantiomeric identification) and 14(*R*),15(*S*)-EpETE (**21**) enantiomers, respectively (Table 2).

On the one hand, these results reveal that *Aae*UPO is a powerful tool to get access to mono-epoxides of n-3 fatty acids with high conversion (>99%), regioselectivity (>90%) and total enantioselectivity (*ee* > 99%) towards the *S*/*R* enantiomers. The same stereoselectivity as observed with P450 BM3, while the opposite enantiomer (*R/S*) is produced by other P450s [49] and plant peroxygenases [14,50].

These stereoselective bioconversions are of interest since recent studies suggest that the bioactivity of oxylipins and derived products can be exerted exclusively by one enantiomer [51,52]. Although the identity of the therapeutically active isomer remains unclear in many diseases, there are some examples of enantiospecific activity reported in the literature. For instance, in addition to the general anti-inflammatory activity displayed by the **15** epoxide, only its *R*/*S* enantiomer has a vasodilatory effect [52], while the *S*/*R* enantiomer has anti-allergy effect [53].

On the other hand, the enzymatic epoxidations with r*Cvi*UPO and its T158F variant did not show such a stereoselectivity (yielding nearly racemic mixtures). Nevertheless, an inversion of the configuration was observed between **21** (56% *R*/*S* by r*Cvi*UPO) and **20** (56% *S*/*R* by the T158F variant), probably induced by the T158F mutation that would affect the fitting of the substrate at the active site.

## 4. Conclusions

When analyzing mono-epoxidation of a series of n-3 and n-6 polyunsaturated fatty acids by several UPOs, high conversions (93%-quant) and regioselectivities (91%-quant) towards the last double bond were accomplished with *Aae*UPO and r*Cvi*UPO (Scheme 1). In the above reactions, *Aae*UPO effectively epoxidize n-3 fatty acids, but it fails to do so with their n-6 counterparts, which, in contrast, are indeed epoxidized by r*Cvi*UPO. Differences in the heme-access channels of these two enzymes seem to lie behind this ability to synthesize n-3 or n-6 mono-epoxides, since the more accessible active site of r*Cvi*UPO allows the substrate to approach the n-6 unsaturation to the heme cofactor.

The regioselectivity of the above reactions was confirmed by 1D and 2D NMR of a selection of mono-epoxide products—17,18-EpEDE (**14**), 17,18-EpETrE (**15**), 19,20-EpDTrE (**17**), 19,20-EpDPE (**18**), 14,15-EpEDE (**20**) and 14,15-EpETE (**21**)—after isolation at mg-scale. Additionally, the total selectivity to *S*/*R* enantiomer of the mono-epoxides from n-3 substrates formed by *Aae*UPO (long UPO family) was demonstrated. Although such stereoselectivity is similar to that of the well-known P450 BM3, UPOs give higher conversion yields and avoid some P450 disadvantages. Contrarily to *Aae*UPO, the reactions with r*Cvi*UPO (short UPO family) pointed out nearly racemic mixtures. The selective synthesis of pure enantiomers of fatty-acid mono-epoxides can shed light on the physiological and pharmacological properties of these compounds.

## Figures and Tables

**Table 1:** Product percentages (relative to total products) and substrate conversions in UPO reactions with n-3 (**1**–**8**) and n-6 (9–11) polyunsaturated fatty acids (see Figures S1 and S2 for chromatographic profiles, and Figure 1 and for substrate, **1**–**11**, and product, **12**–**22**, formulae).

Substrate	Enzyme	Mono- Epoxide Production ^1^	Mono- Epoxide Formulae	Other Epoxides ^2^	Hydroxy Fatty Acids	Conversion
**HTA (1)**	*Aae*UPO	97%	**12**	-	3%	>99%
**SDA (2)**	*Aae*UPO	>99%	**13**	-	-	>99%
**ETE (3)**	*Aae*UPO	93%	**14**	6%	1%	>99%
**EPA (4)**	*Aae*UPO	95%	**15**	5%	-	>99%
**HPA (5)**	*Aae*UPO	96%	**16**	-	4%	>99%
**DPA (6)**	*Aae*UPO	91%	**17**	9%	-	>99%
**DHA (7)**	*Aae*UPO	94%	**18**	6%	-	>99%
**Nisinic (8)**	*Aae*UPO	91%	**19**	9%	-	>99%
**DGLA (9)**	r*Cvi*UPO	74%	**20**	19%	7%	96%
**DGLA (9)**	F88L	51%	**20**	48%	1%	95%
**DGLA (9)**	T158F	92%	**20**	-	8%	93%
**AA (10)**	r*Cvi*UPO	92%	**21**	-	8%	94%
**AA (10)**	F88L	74%	**21**	25%	1%	99%
**AA (10)**	T158F	95%	**21**	-	5%	57%
**AdA (11)**	r*Cvi*UPO	74%	**22**	19%	7%	>99%
**AdA (11)**	F88L	20%	**22**	80%	-	>99%
**AdA (11)**	T158F	95%	**22**	-	5%	94%

^1^ Mono-epoxides are located at the last double bond. ^2^ Other epoxides include hydroxy-epoxides and di-epoxides from the n-3 and n-6 fatty acids, respectively.

**Table 2:** Stereoselectivity analysis of mono-epoxides produced by UPOs (see Figure 7 and Figure S12 for HPLC profiles).

Substrate	Enzyme	Epoxide Product	Main Enantiomer
**ETE (3)**	*Aae*UPO	17,18-EpEDE (**14**)	>99% *S*/*R* ^1^
**EPA (4)**	*Aae*UPO	17,18-EpETrE (**15**)	>99% *S*/*R*
**DPA (6)**	*Aae*UPO	19,20-EpDTrE (**17**)	>99% *S*/*R*
**DHA (7)**	*Aae*UPO	19,20-EpDPE (**18**)	>99% *S*/*R*
**DGLA (9)**	T158F	14,15-EpEDE (**20**)	56% *S/R* ^1^
**AA (10)**	r*Cvi*UPO	14,15-EpETE (**21**)	56% *R*/*S*

^1^ Assigned following the elution order of related epoxides since no standards are available.

## Data Availability

Data are contained within the article.

## Supplementary Materials

The following are available online (at https://www.mdpi.com/article/10.3390/antiox10121888/s1) includes: Figure S1: Fragmentation pattern of n-3 mono-epoxides (Scheme S1); Selective epoxidation of eight n-3 fatty acids by *Aae*UPO; Figure S2: Selective epoxidation of three n-6 fatty acids by r*Cvi*UPO and its variants; Figure S3: ^1^H and ^13^C-NMR characterization of 17,18-EpEDE (14); Figure S4: ^1^H and ^13^C-NMR characterization of 17,18-EpETrE (15); Figure S5: ^1^H and ^13^C-NMR characterization of 19,20-EpDTrE (17); Figure S6: ^1^H and ^13^C-NMR characterization of 19,20-EpDPE (18); Figure S7: ^1^H and ^13^C-NMR characterization of 14,15-EpEDE (20); Figure S8: ^1^H and ^13^C-NMR characterization of 14,15-EpETE (21); Figure S9: HMBC spectra of 17,18-EpEDE (14) and 17,18-EpETrE (15); Figure S10: HMBC spectra of 19,20-EpDTrE (17) and 14,15-EpEDE (20); Figure S11: Sections of the *Cvi*UPO and *in-silico* mutated F88L and T158F molecules; Figure S12: Chiral HPLC analyses of isolated n-3 and n-6 mono-epoxides.
