# Supplementary material for: Regioselective and Stereoselective Epoxidation of n-3 and n-6 Fatty Acids by Fungal Peroxygenases
Source: Antioxidants (Basel). 2021 Nov 25;10(12):1888. doi: 10.3390/antiox10121888 (PMC8698580; doi:10.3390/antiox10121888)
Supplement: Supplementary file 1 [file antioxidants-10-01888-s001.zip › antioxidants-1448549-supplementary.pdf]

antioxidants

SUPPLEMENTARY MATERIALS

Regioselective and stereoselective epoxidation of n-3 and n-6 fatty acids by fungal peroxygenases

Alejandro González-Benjumea [1], Dolores Linde [2], Juan Carro [2], René Ullrich [3], Martin Hofrichter [3], Angel T. Martínez [2,*] and Ana Gutiérrez [1,*]

[1] Instituto de Recursos Naturales y Agrobiología de Sevilla (IRNAS), Consejo Superior de Investigaciones Científicas (CSIC), Reina Mercedes 10, E-41012, Seville, Spain; a.g.benjumea@irnas.csic.es (A.G.-B.); anagu@irnase.csic.es (A.G.)
[2] Centro de Investigaciones Biológicas "Margarita Salas" (CIB), CSIC, Ramiro de Maeztu 9, E-28040, Madrid, Spain; jcarro@cib.csic.es (J.C.); lolalinde@cib.csic.es (D.L.); atmartinez@cib.csic.es (A.T.M.)
[3] Technische Universität Dresden, International Institute Zittau (IHIZ), Markt 23, D-02763, Zitau, Germany; rene.ullrich@tu-dresden.de (R.U.); martin.hofrichter@tu-dresden.de (M.H.)
* Correspondence: anagu@irnase.csic.es; Tel.: +34 954624711; atmartinez@cib.csic.es; Tel.: +34 918373112

This Supplementary Materials includes: Fragmentation pattern of n-3 mono-epoxides (**Scheme S1**); Selective epoxidation of eight n-3 fatty acids by *Aae*UPO (**Figure S1**); Selective epoxidation of three n-6 fatty acids by r*Cvi*UPO and its variants (**Figure S2**); ^{1}H and ^{13}C-NMR characterization of 17,18-EpEDE (**14**) (**Figure S3**); ^{1}H and ^{13}C-NMR characterization of 17,18-EpETrE (**15**) (**Figure S4**); ^{1}H and ^{13}C-NMR characterization of 19,20-EpDTrE (**17**) (**Figure S5**); ^{1}H and ^{13}C-NMR characterization of 19,20-EpDPE (**18**) (**Figure S6**); ^{1}H and ^{13}C-NMR characterization of 14,15-EpEDE (**20**) (**Figure S7**); ^{1}H and ^{13}C-NMR characterization of 14,15-EpETE (**21**) (**Figure S8**); HMBC spectra of 17,18-EpEDE (**14**) and 17,18-EpETrE (**15**) (**Figure S9**); HMBC spectra of 19,20-EpDTrE (**17**) and 14,15-EpEDE (**20**) (**Figure S10**); Sections of the *Cvi*UPO and *in-silico* mutated F88L and T158F molecules (**Figure S11**); Chiral HPLC analyses of isolated n-3 and n-6 mono-epoxides (**Figure S12**).

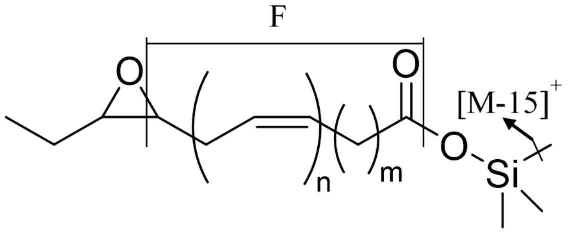

Scheme S1. Fragmentation pattern of n-3 mono-epoxides (**Figure S1** compounds **12-19**) yielding characteristic fragment *F* during GC-MS analysis as TMSi derivatives (*m*, 3-9; and *n*, 2-5).

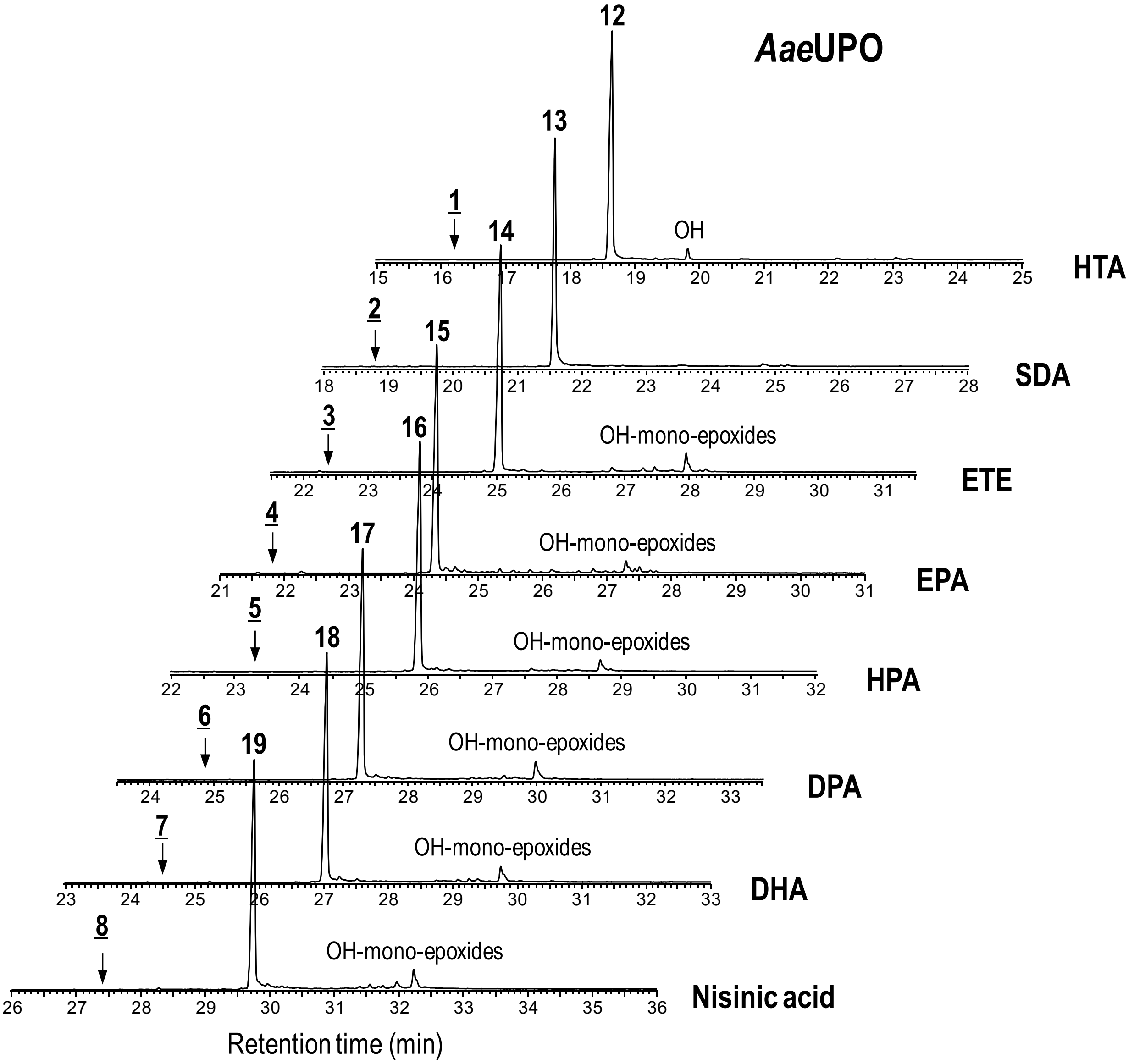

Figure S1. Selective epoxidation of eight n-3 polyunsaturated fatty acids (<u>1</u>-<u>8</u>) by *Aae*UPO yielding the terminal mono-epoxides **12-19** (91% to >99% of all products) and minor amounts of hydroxy and hydroxy-epoxy derivatives.

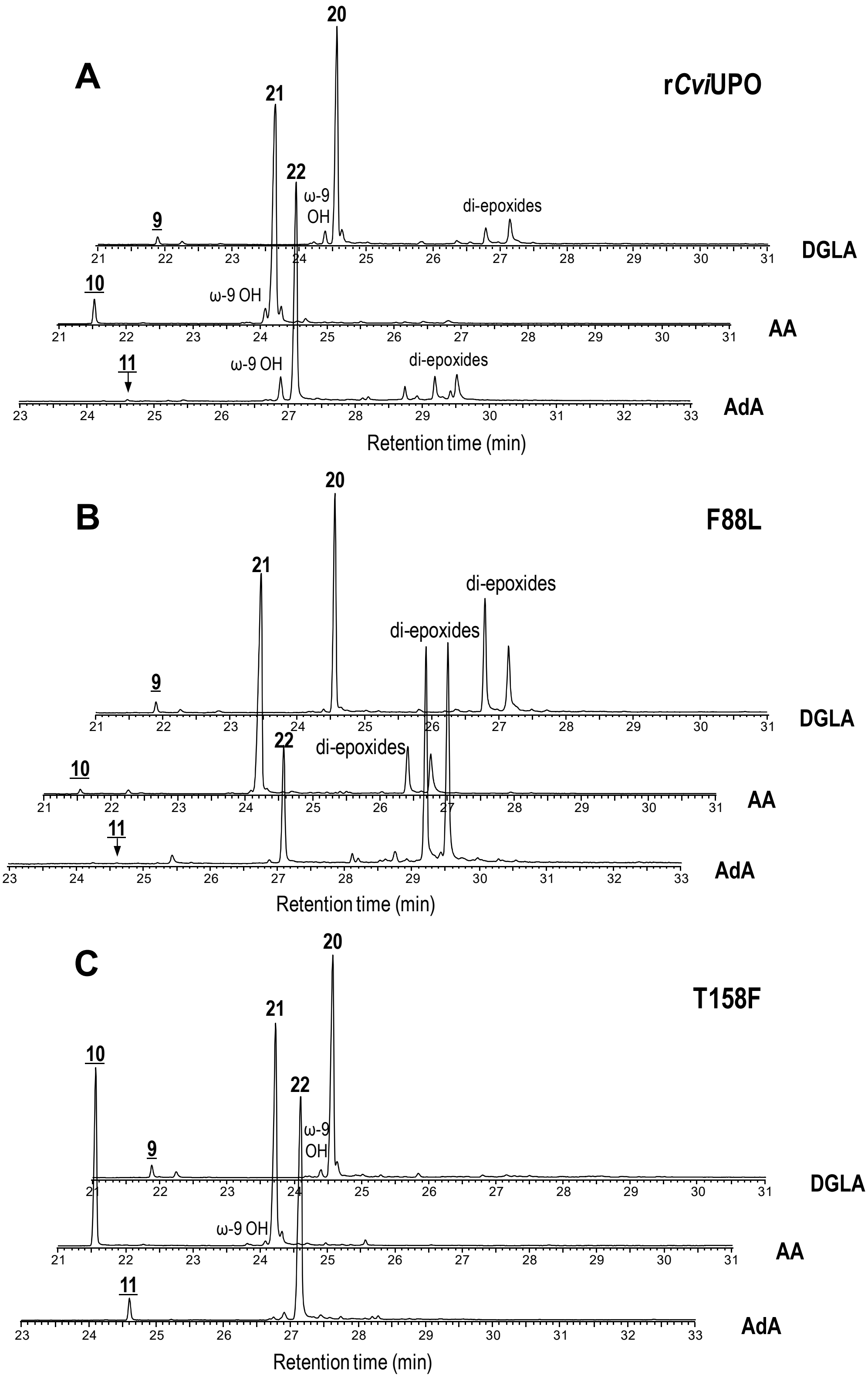

Figure S2. Selective epoxidations of three n-6 polyunsaturated fatty acids (**9**-**11**) by r*Cvi*UPO (**A**) and its F88L (**B**) and T158F (**C**) variants. Terminal mono-epoxides (**20-22**) represented 92-95% of all products in the T158F reactions, while di-epoxides (up to 80%) are produced by F88L, and small di-epoxide (up to 19%) and hydroxyfatty acid (up to 8%) amounts are formed by r*Cvi*UPO.

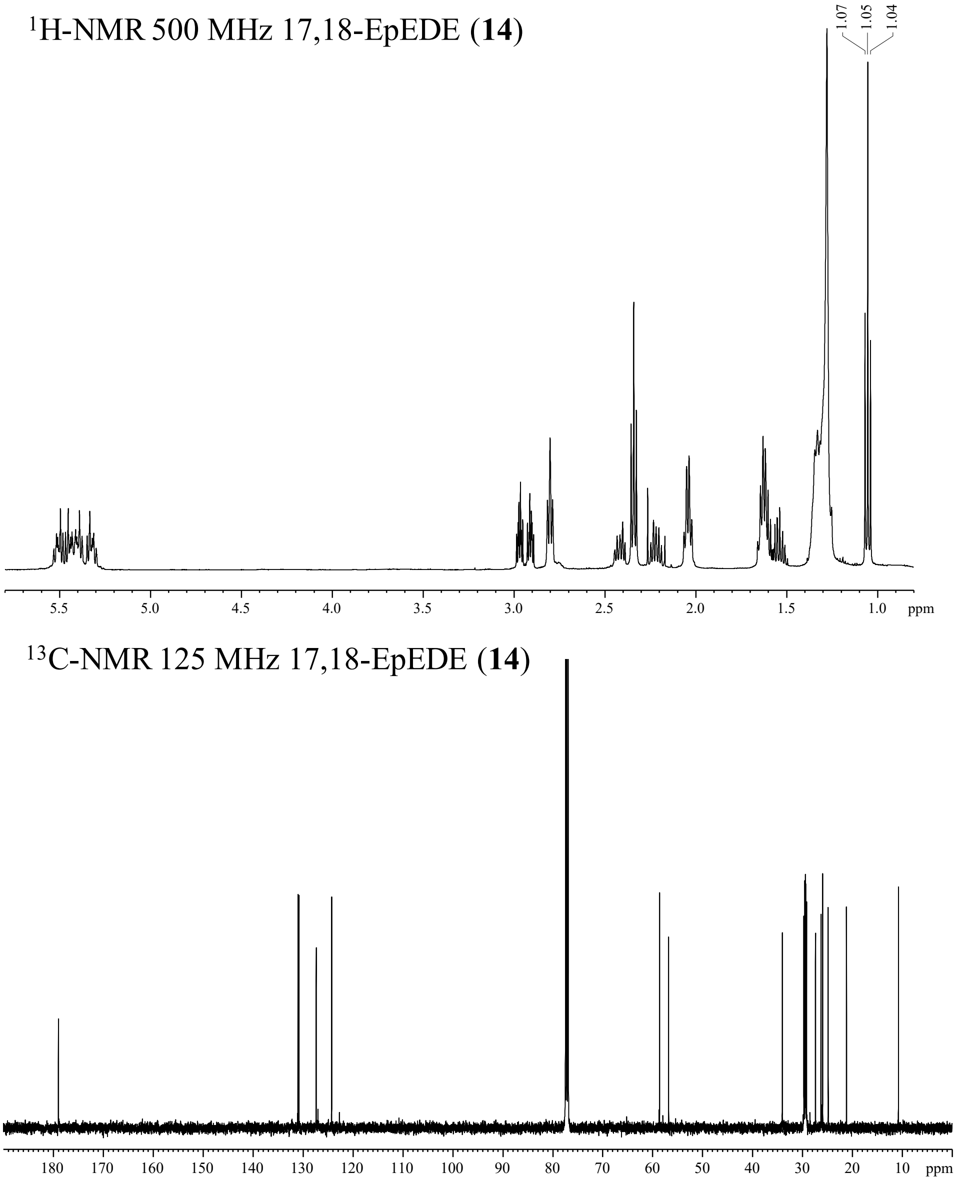

Figure S3. ^{1}H and ^{13}C-NMR characterization of 17,18-EpEDE (**14**) from ETE (**3**) reaction with *Aae*UPO.

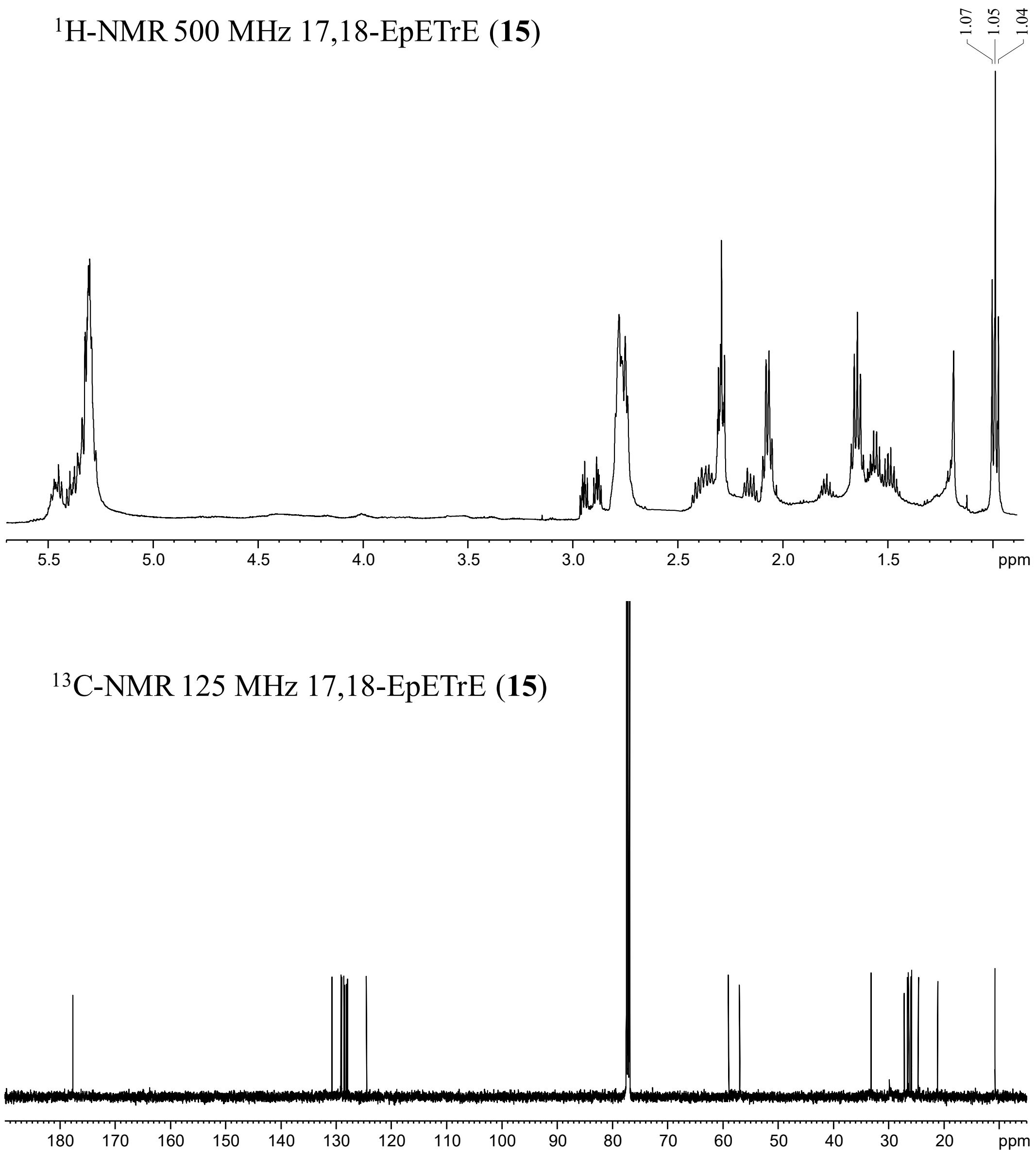

Figure S4. ^{1}H and ^{13}C-NMR characterization of 17,18-EpETrE (**15**) from EPA (**4**) reaction with *Aae*UPO.

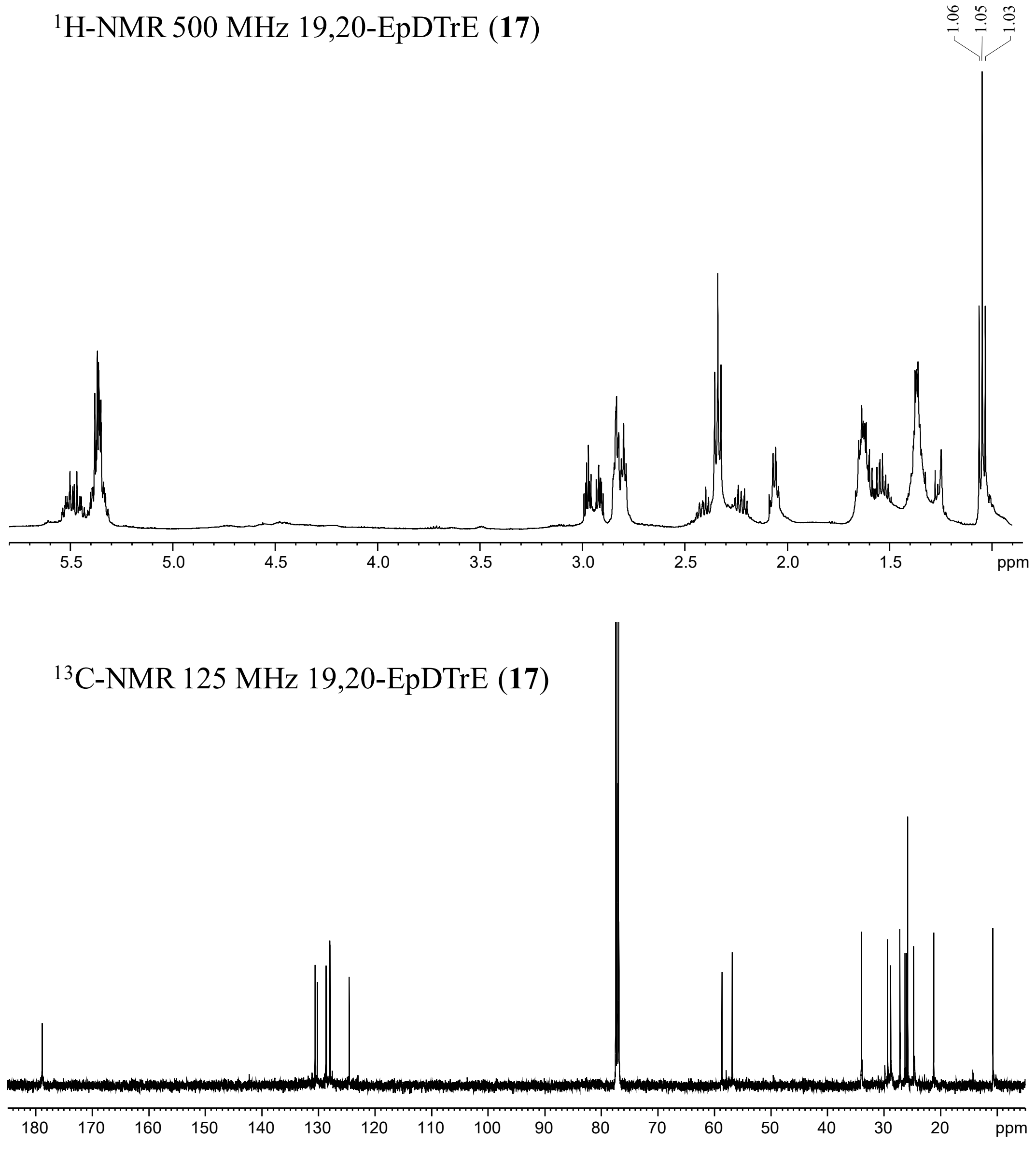

Figure S5. ¹H and ¹³C-NMR characterization of 19,20-EpDTrE (**17**) from DPA (**6**) reaction with *Aae*UPO.

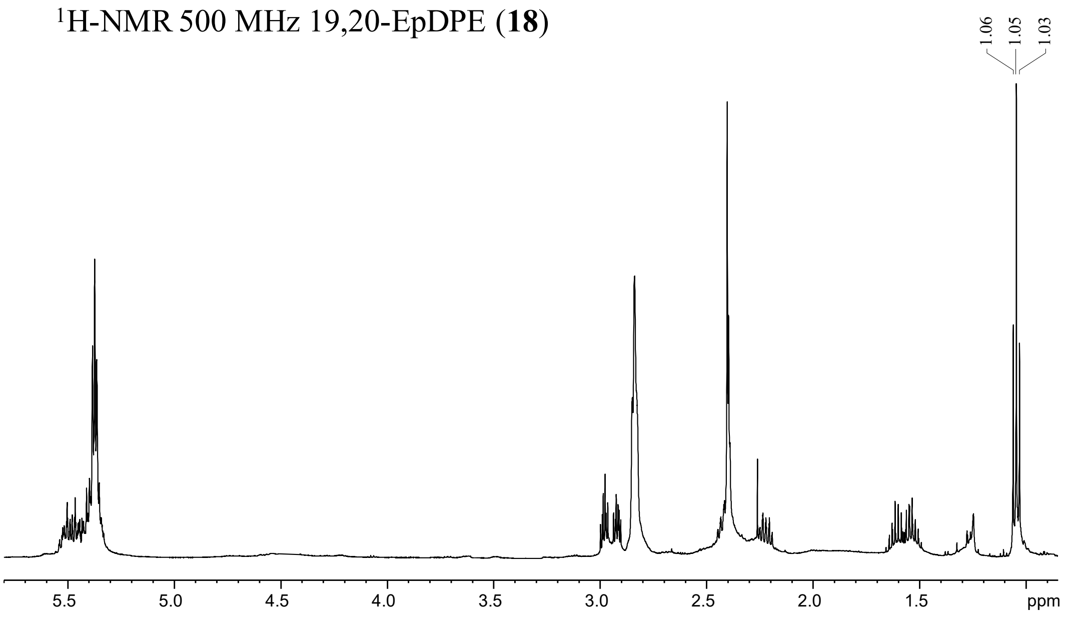

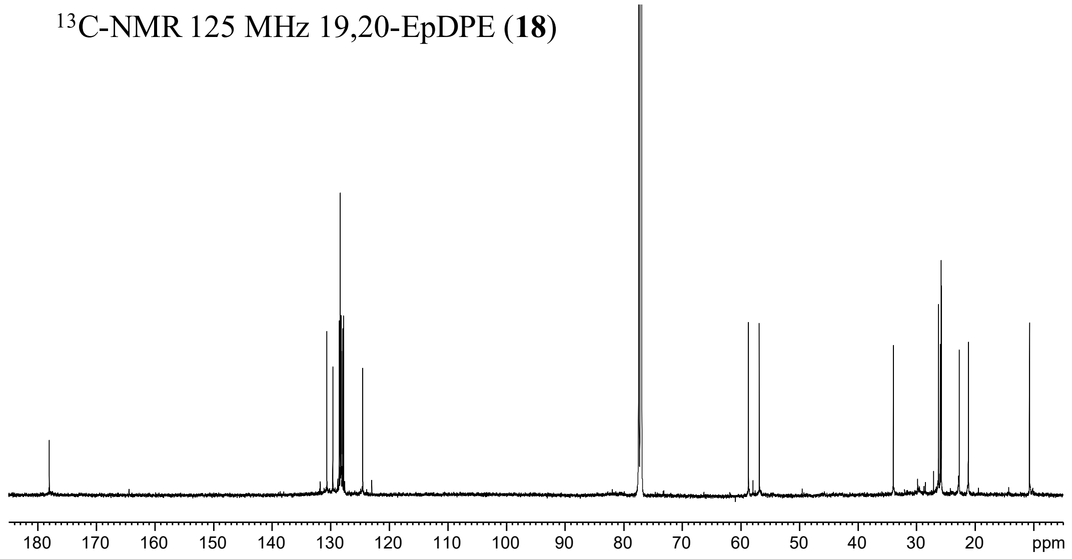

Figure S6. ^{1}H and ^{13}C-NMR characterization of 19,20-EpDPE (**18**) from DHA (**7**) reaction with *Aae*UPO.

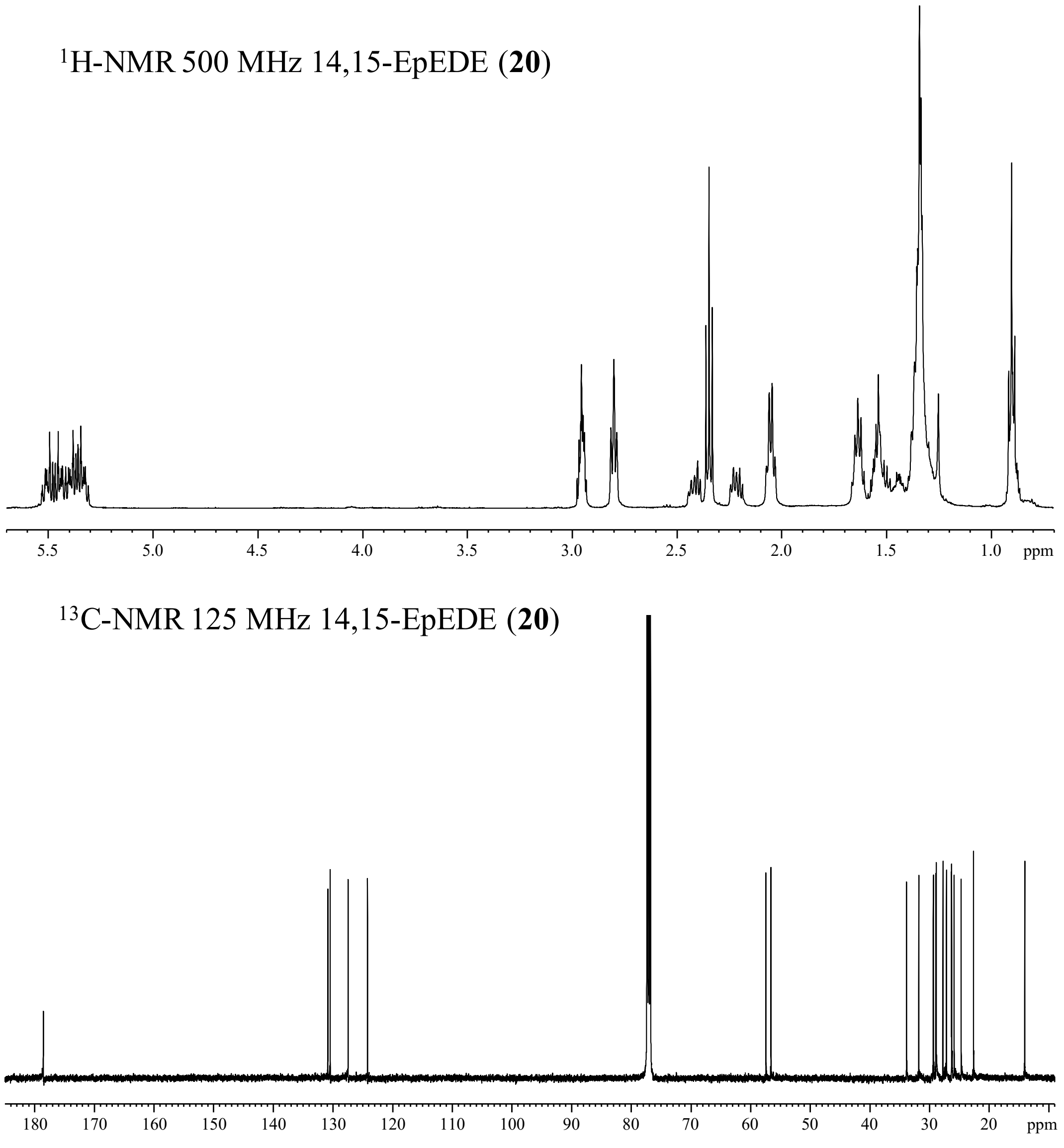

Figure S7. ^{1}H and ^{13}C-NMR characterization of 14,15-EpEDE (**20**) from DGLA (**9**) reaction with the T158F variant of r*Cvi*UPO.

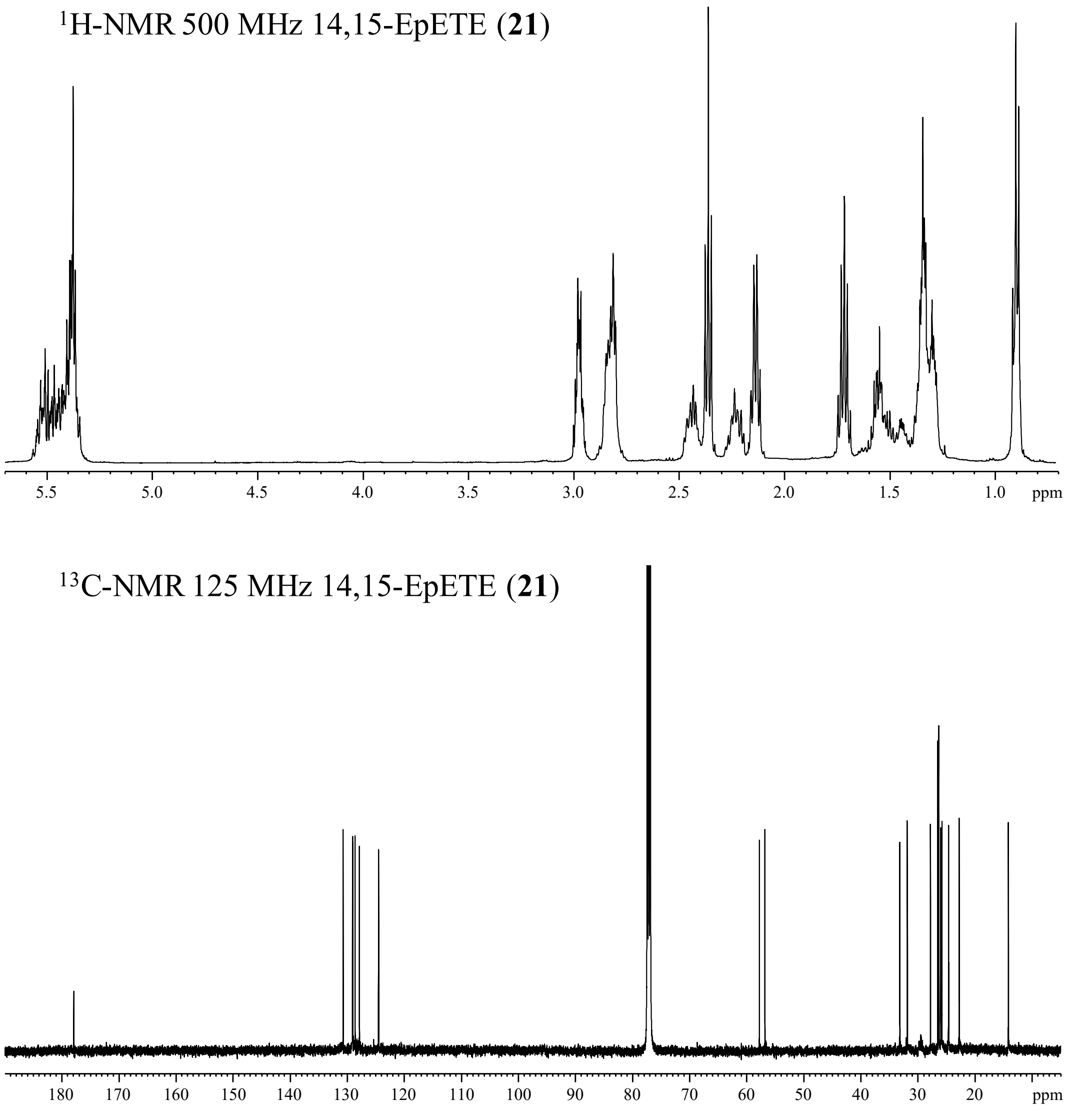

Figure S8. ^{1}H and ^{13}C-NMR characterization of 14,15-EpETE (**21**) from AA (**10**) reaction with r*Cvi*UPO.

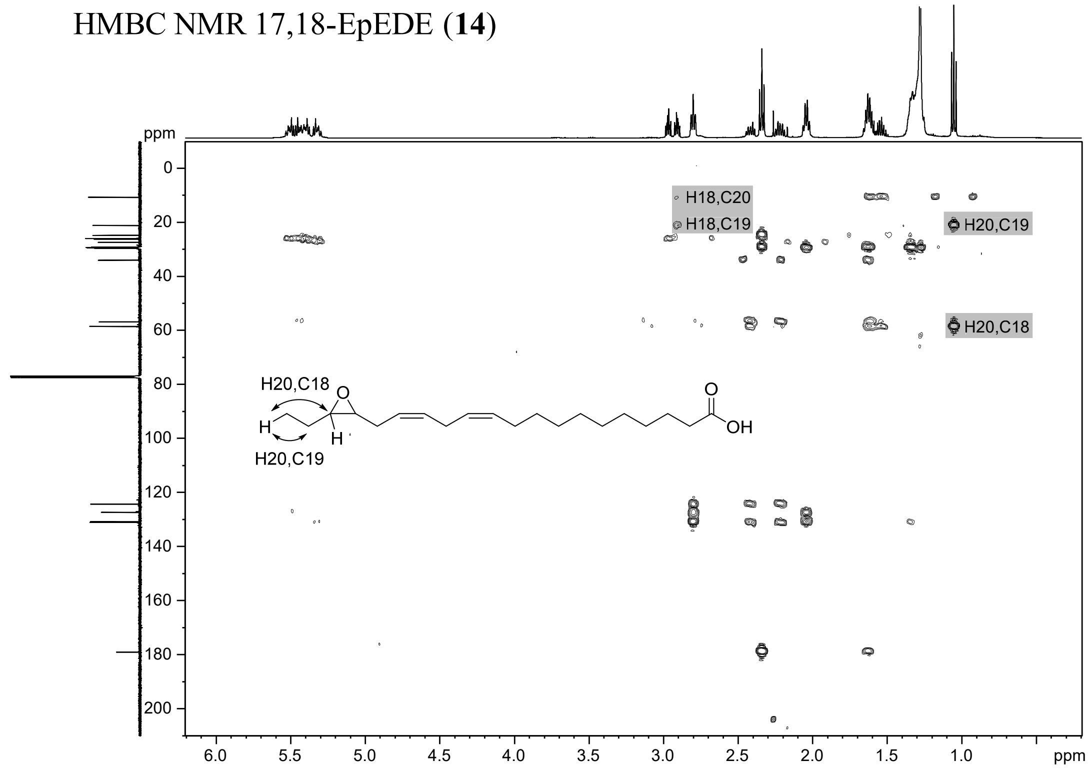

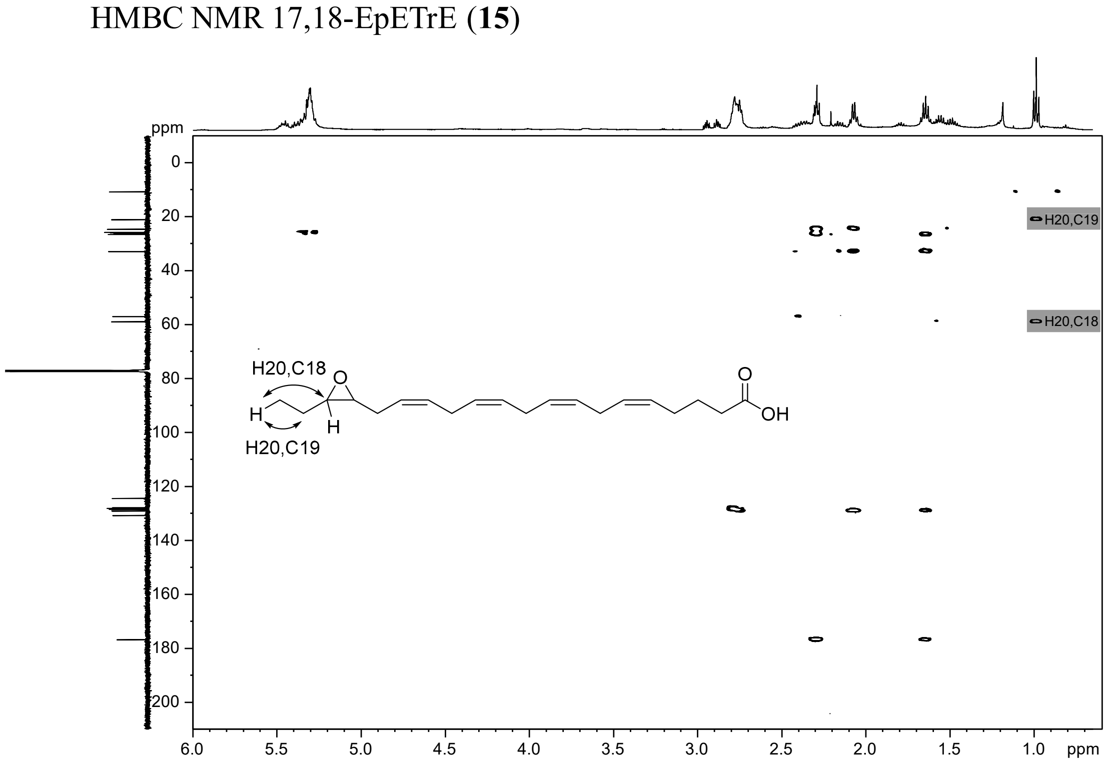

Figure S9. HMBC 2D-NMR spectra of 17,18-EpEDE (**14**) from ETE (**3**) reaction with *Aae*UPO; and 17,18-EpETrE (**15**) from EPA (**4**) reaction with with *Aae*UPO. Several relevant correlation signals are indicated.

HMBC NMR 19,20-EpDTrE (**17**)

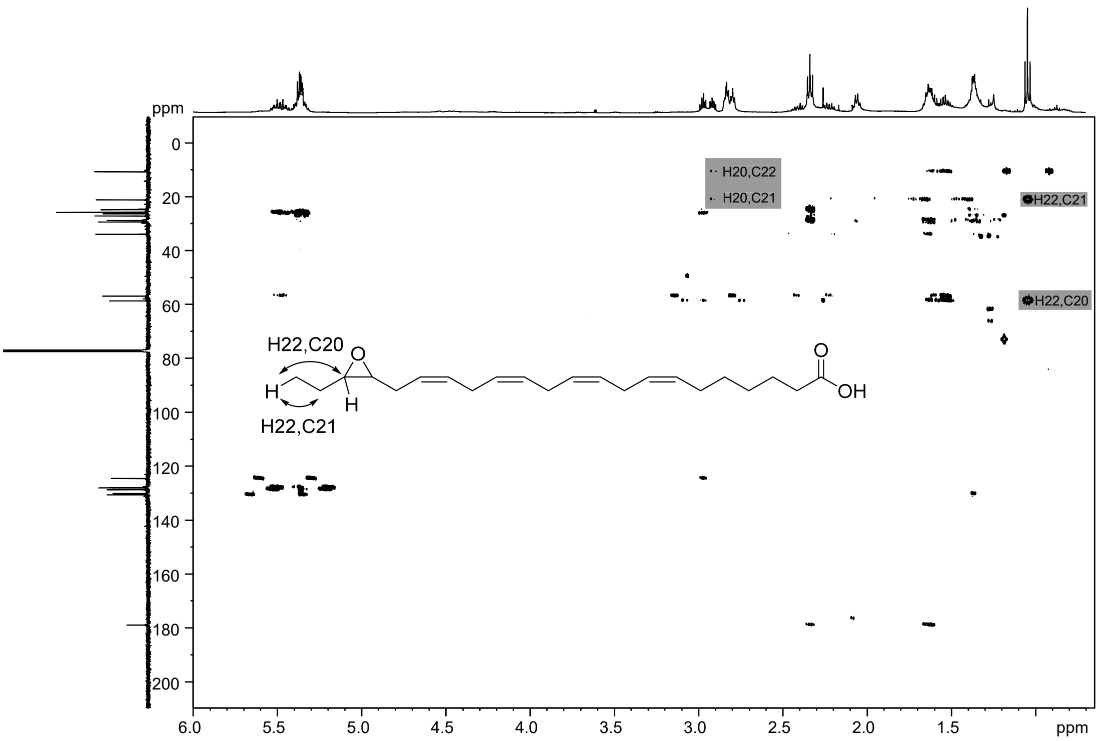

HMBC NMR 14,15-EpEDE (**20**)

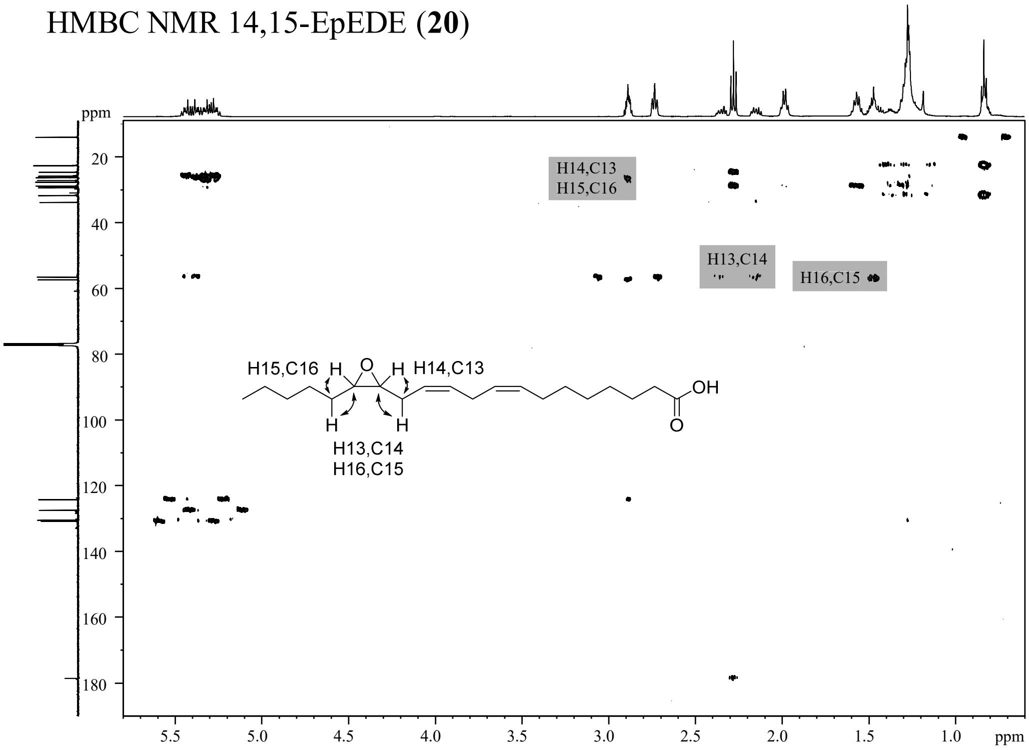

Figure S10. HMBC 2D-NMR spectra of 19,20-EpDTrE (**17**) from DPA (**6**) reaction with *Aae*UPO; and 14,15-EpEDE (**20**) from DGLA (**9**) reaction with the T158F variant of r*Cvi*UPO. Several relevant correlation signals are indicated.

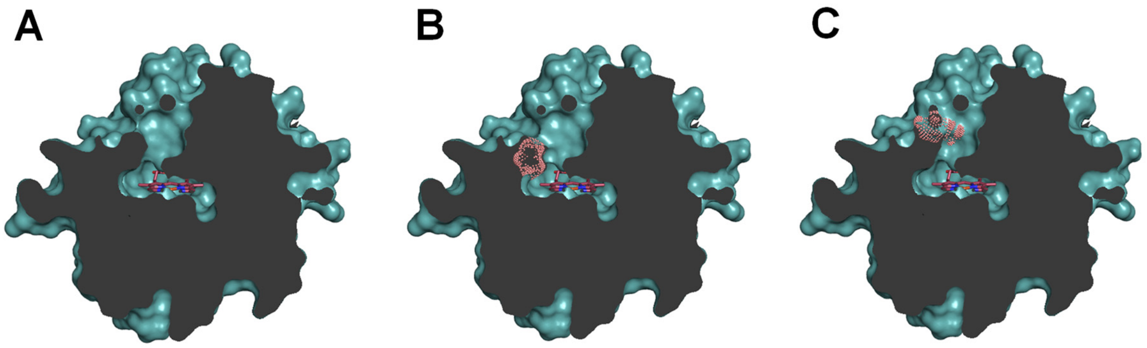

Figure S11. Sections of the *Cvi*UPO (**A**) and *in-silico* mutated F88L (**B**) and T158F (**C**) molecules, illustrating differences in the heme-access channels with the mutated residues highlighted as salmon dots in **B** (Leu) and **C** (Phe). External surface of the protein and surface of the residues lining the channel are shown in light teal, and the buried heme is displayed as sticks in CPK coloring. Structures are based on an homology model constructed for *Cvi*UPO.

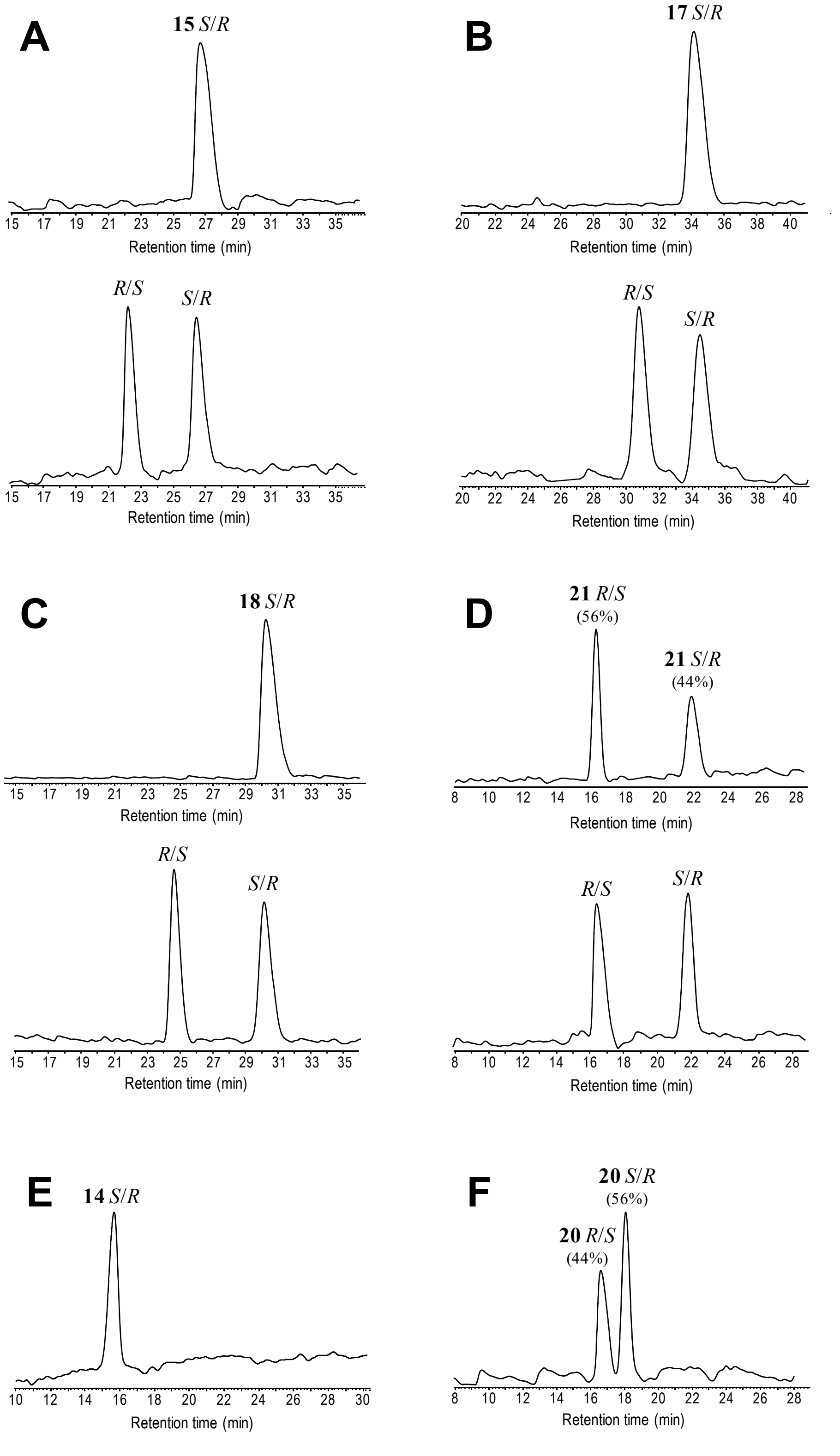

Figure S12. Chiral HPLC analyses of isolated n-3 and n-6 mono-epoxides: **A)** 17(*S*),18(*R*)-EpETrE (**15**) from *Aae*UPO reaction with EPA (**4**) (*top*) compared with a racemic standard (*bottom*); **B)** 19(*S*),20(*R*)-EpDTrE (**17**) from *Aae*UPO reaction with DPA (**6**) (*top*) compared with a racemic standard (*bottom*); **C)** 19(*S*),20(*R*)-EpDPE (**18**) from *Aae*UPO reaction with DHA (**7**) (*top*) compared with a racemic standard (*bottom*); **D)** 14(*R*),15(*S*)-EpETE and 14(*S*),15(*R*)-EpETE (**21**) from r*Cvi*UPO reaction with AA (**10**) (*top*) compared with a racemic standard (*bottom*); **E)** 17(*S*),18(*R*)-EpEDE (**14**) from *Aae*UPO reaction with ETE (**3**); **F** 14(*R*),15(*S*)-EpEDE and 14(*S*),15(*R*)-EpEDE (**20**) from the variant T158F of r*Cvi*UPO reaction with DGLA (**9**) (no standards available for **E** and **F**). Profiles were recorded at 202 nm (**B-F**) or 204 nm (**A**).
